# Thinking outside the shoulder: A systematic review and metanalysis of kinetic chain characteristics in non-athletes with shoulder pain

**DOI:** 10.1371/journal.pone.0314909

**Published:** 2024-12-09

**Authors:** Hilmaynne Renaly Fonseca Fialho, Vander Gava, Romário Nóbrega Santos Fonseca, Danilo Harudy Kamonseki, Germanna Medeiros Barbosa

**Affiliations:** 1 Post-Graduate Program in Rehabilitation Sciences, School of Health Sciences of Trairi, Federal University of Rio Grande do Norte, Santa Cruz, RN, Brazil; 2 Department of Physical Therapy, Post-Graduate Program in Physical Therapy, Federal University of São Carlos, São Carlos, SP, Brazil; 3 Department of Physical Therapy, Federal University of Paraíba, João Pessoa, PB, Brazil; Iran University of Medical Sciences, ISLAMIC REPUBLIC OF IRAN

## Abstract

**Introduction:**

The kinetic chain is important in the proximal-distal energy transfer along body segments. Although biomechanical impairments in components of the kinetic chain have already been reported in athletes with shoulder pain, such investigations in non-athlete individuals have not yet been synthesized.

**Objective:**

To systematically review the literature and analyze the quality of evidence on the characteristics of the kinetic chain of non-athletes with shoulder pain compared to asymptomatic individuals.

**Methods:**

Studies published until February 2024 (without language restrictions) that quantitatively assessed outcomes related to the kinetic chain of non-athlete individuals were searched in five databases (MEDLINE, CINAHL, Web of Science, EMBASE, and SCOPUS). The risk of bias and quality of evidence were analyzed using the Joanna Briggs Institute Critical Appraisal Tool for Analytical Cross-Sectional Studies and the Grading of Recommendations, Assessment, Development, and Evaluation approach, respectively. To summarize the findings, meta-analyses with random-effects models were performed.

**Results:**

Six cross-sectional studies (n = 562 [282 with shoulder pain], mean age = 48.7 ± 8.1 years) with low risk of bias were included in this review. Very low-quality evidence suggests that individuals with shoulder pain may present a lower active range of motion and muscular endurance/strength in the cervical spine, thoracolumbar region, and hip, as well as deficits in neuromuscular control of the lower extremities. Findings related to thoracic spine posture were conflicting and no differences were found in cervical spine isometric muscular strength between individuals with and without shoulder pain.

**Conclusion:**

Individuals with shoulder pain may present alterations in active mobility, muscular endurance, and neuromuscular control in kinetic chain segments. These findings suggest that broader physical assessments considering components of the kinetic chain may be clinically relevant in this population. However, based on the very low quality of evidence, the findings of this review should be interpreted with caution.

## Introduction

Shoulder pain is one of the most common musculoskeletal complaints in the general population, with an overall prevalence of up to 55% and an estimated incidence of 62 cases per 1000 persons/year [1]. This condition is associated with functional impairments that can negatively affect quality of life and limit participation in activities of daily living and work [2,3]. Biomechanical alterations in the shoulder complex, such as deficits in range of motion (ROM) and imbalances in muscular activation or strength, are frequently investigated and observed in individuals with shoulder pain [4]. However, it remains unclear whether alterations along the kinetic chain may be also present in this population [5].

The kinetic chain concept suggests a complex interaction and coordination of different body segments sequentially activated that generate and transfer energy in a proximal-distal pattern during functional movements [6]. Most studies [7–15] that explored the biomechanical characteristics of the kinetic chain were conducted in athletes. They reported that impairments in its components may overload the upper extremities, increasing the risk of injury and/or pain in this region and reducing performance during sports practice. Likewise, a recent systematic review [16] reported that alterations in stability and/or muscular function (i.e., strength, endurance, and/or power) in the trunk and lower extremities may be related to shoulder complaints in athletes of throwing sports.

Outside the sporting context, the available evidence regarding the characteristics of the kinetic chain of individuals with shoulder pain is still scarce. While considering that muscles generally do not act independently during functional movements [17], alterations in segments of the kinetic chain–such as deficits in muscular function and/or ROM–can jeopardize the corporal transmission of energy necessary for daily activities [6]. Understanding the characteristics of the kinetic chain of individuals with shoulder pain can be important not only for better guiding the assessment and treatment of the shoulder but also for clinical and scientific reasoning for future investigations of encompassing additional factors beyond the shoulder itself in rehabilitation.

Therefore, this study aimed to systematically review the literature that investigated the characteristics of segments of the kinetic chain of non-athlete individuals with and without shoulder pain.

## Materials and methods

This systematic review is reported according to the Preferred Reporting Items for Systematic Reviews and Meta-Analysis (PRISMA) [18] (checklist available in S1 File). The review protocol was prospectively registered on PROSPERO (CRD42022384459).

### Search methods for identification of studies

A systematic literature search was conducted in MEDLINE/PubMed (National Library of Medicine), Cumulative Index to Nursing and Allied Health Literature (CINAHL Full Text [EBSCO]), Web of Science (Clarivate), EMBASE (Elsevier), and Scopus (Elsevier) databases from inception up to February 2024. Medical Subject Headings (MeSH) terms and/or keywords regarding shoulder pain and kinetic chain were combined and adapted for each database. No date or language restrictions were applied. The keywords related to shoulder, kinetic chain, and physical assessment were used in the searches. The detailed search strategies are summarized in the S2 File.

### Eligibility criteria

The eligibility criteria were defined based on the PICOS (acronym for Population, Intervention, Comparison, Outcomes, and Study type) strategy:[19]

Population–studies that assessed non-athletic individuals (age ≥ 18 years) with shoulder pain.

Intervention–no specific criteria were considered for case-control/ observational studies. Therapeutic exercises (e.g., strengthening, stretching, motor control) were considered for clinical trials.

Comparison–studies that assessed non-athletic individuals (age ≥ 18 years) without shoulder pain were considered for case-control. No interventions or other interventions besides therapeutic exercises were considered for clinical trials.

Outcomes–studies that quantitatively measured at least one of the following outcomes: posture, range of motion, muscular function, or balance/stability/neuromuscular control of kinetic chain components.

Study type–Any study type, except unpublished reports, conference abstracts, book chapters, and/or studies involving subjects with traumatic shoulder pain, history of shoulder surgery, diagnosis of glenohumeral instability, or frozen shoulder, was considered.

### Study selection

The studies identified during the literature search were imported into the Systematic Review Accelerator automation tool [20], and duplicates were analyzed and excluded. The remaining studies were screened by two independent reviewers (HRFF and VG) to exclude those that did not fit the inclusion criteria. After screening titles and abstracts, full texts were analyzed and their reference lists were screened for potentially relevant publications not retrieved during searches. The selection process was conducted by consensus, and a third reviewer (DHK) was consulted in case of discrepancies.

### Data extraction

Two reviewers (VG and RNSF) independently extracted the data and a third reviewer (HRFF) verified it in case of discrepancies. A standardized form was used to extract data such as identification, methodological design, and relevant information regarding the sample, outcomes, and quantitative results of each included study (S3 File). When necessary, the reviewers arithmetically converted values of median and range into mean and standard deviation [21].

### Risk of bias

Two reviewers (VG and RNSF) independently evaluated the risk of bias in the included studies using the Joanna Briggs Institute (JBI) Critical Appraisal Tool for Analytical Cross-Sectional Studies [22]. This 8-item checklist relies on the subjective appraisal of the reviewer and does not generate a final score. In cases of discrepancy regarding the feasibility of including the study, a third reviewer (HRFF) was consulted.

### Data synthesis

A narrative synthesis with a qualitative summary strategy was conducted. In order to summarize the kinetic chain characteristics of non-athletes with and without shoulder pain, meta-analyses were carried out using the RevMan 5.4 software (© The Cochrane Collaboration, London, UK). Means and standard deviations were extracted from the results of each included study. Mean differences or standardized mean differences (when different methods assessed the outcomes of two or more studies) were calculated using a random-effects model in the meta-analysis [23].

The quality of evidence was determined using the Grading of Recommendations, Assessment, Development, and Evaluation (GRADE) approach [24,25]. Each included study is assessed by the GRADE approach according to five domains:

Risk of bias–the quality of the evidence was downgraded by one level if more than 25% of the studies in each comparison presented a high risk of bias and by two levels if more than 50% of the studies presented a high risk of bias.

Inconsistency–the quality of the evidence was downgraded by one level if significant statistical heterogeneity was observed in the results (p < 0.05 and I^2^ test > 50%) and by two levels in case of serious statistical heterogeneity (p < 0.05 and I^2^ test > 75%).

Indirectness–the quality of the evidence was downgraded in one level when clinical heterogeneity was observed between studies (patients, assessments, or outcome measures).

Imprecision–the quality of the evidence was downgraded by one level if the pooled sample was smaller than 200 individuals or a wide confidence interval was observed (95% confidence interval crossing the 0.5 mark in any direction) and downgraded by two levels when both criteria were met.

Publication bias–the quality of the evidence was downgraded if small studies were sponsored or the investigators stated conflicts of interest.

After assessing the required domains, the quality of evidence is presented as high, moderate, low, or very low:

High–the true effect lies close to that of the estimate of the effect.

Moderate–the true effect is likely to be close to the estimate of the effect.

Low–the true effect may be substantially different from the estimate of the effect.

Very low–the true effect is likely to be substantially different from the estimate of the effect.

## Results

### Study selection, risk of bias, and quality assessment

**Fig 1:**
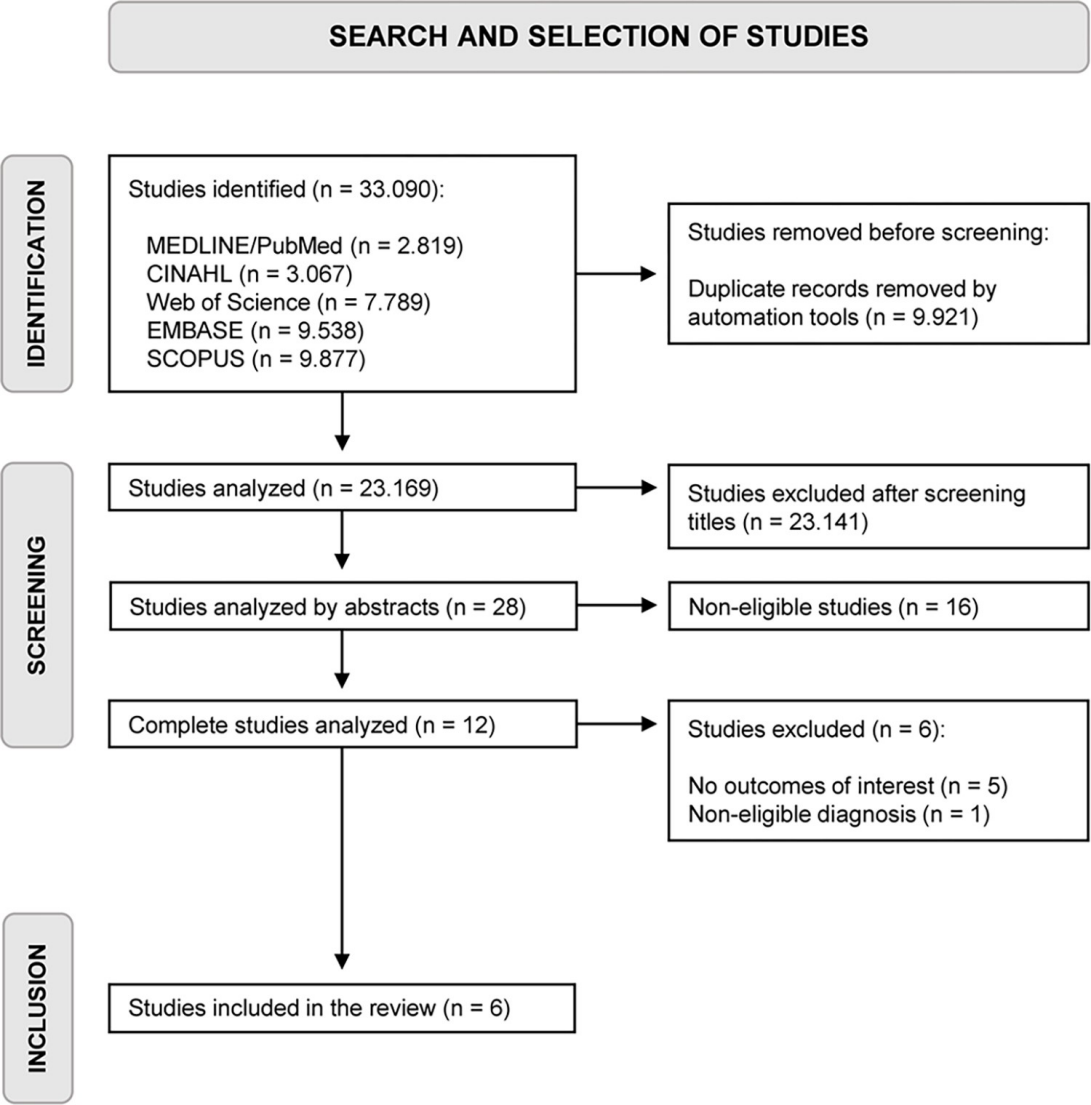
Flow diagram of the review.

The initial search retrieved 33.090 studies and, after duplicate removal, 20.549 studies remained. The full text of 12 cross-sectional studies was assessed and six studies [5,26–30] were included in this review (Fig 1).

Characteristics and results of each included study are presented in Table 1. The pooled sample was 562 individuals (282 with shoulder pain), with an average age of 48.7 ± 8.1 years. Approximately 50% of the pooled sample was women and the main diagnosis of shoulder pain was subacromial impingement syndrome. The following outcomes of interest were assessed by the studies: active ROM (i.e., cervical spine, thoracolumbar region, and hip), thoracic spine posture, isometric muscular strength (i.e., cervical spine), and endurance (i.e., thoracolumbar region and hip), and neuromuscular control of the lower extremities.

**Table 1:** Characteristics of the included studies.

Authors, publication year	Country, language	Study design	Diagnose/population	Control group	Assessments	Total sample characteristics^a^
Meurer et al., 2004 [28]	Germany, German	Cross-sectional	Subacromial impingement syndrome (≥ 3 months) with (n = 23) and without (n = 27) tendinosis calcarea diagnosed by clinical tests (Jobe, Neer, and painful arc) and imaging examinations, respectively	Individuals without evidence of shoulder pathologies (self-report)	*Inclinometry (seated)*^*b*^ · Static thoracic posture · Thoracolumbar spine active ROM on the sagittal plane · Thoracolumbar spine active ROM on the frontal plane · Thoracolumbar spine active ROM on the transverse plane	n = 100 (50 with pain) Age^c^ = 50.1 ± 6.9 Women = 57%
Theisen et al., 2010 [27]	Germany, English	Cross-sectional	Subacromial impingement syndrome diagnosed by clinical tests (Neer, Hawkins-Kennedy, Speed, and supraspinatus muscle test) and imaging examinations	Individuals without evidence of shoulder pathologies (self-report, clinical and imaging examination)	*Ultrasound topometric measurements (seated)* · Static thoracic posture · Thoracolumbar spine flexion active ROM · Thoracolumbar spine extension active ROM · Thoracolumbar spine active ROM on the sagittal plane	n = 78 (39 with pain) Age^c^ = 56.2 ± 8.2 Women = 41%
Hunter et al., 2020 [26]	Australia, English	Cross-sectional	Subacromial impingement syndrome (≥ 3 months) diagnosed by imaging examination	Individuals without evidence of shoulder pathologies (imaging examination)	*Radiographic imaging*: *modified Cobb angle (standing)* · Static thoracic posture *Inclinometry (seated)* · Thoracolumbar spine flexion active ROM · Thoracolumbar spine extension active ROM · Thoracolumbar spine active ROM on the sagittal plane	n = 78 (39 with pain) Age = 56.4 ± 10.8 Women = 50%
Maciel & Sousa, 2022 [5]	Brazil, English	Cross-sectional	Unilateral shoulder pain (≥ 6 months) and pain at rest ≥ 2 in the Penn Shoulder Score Questionnaire	Individuals without shoulder pain	*Star Excursion Balance Test* · Neuromuscular control of the lower extremities *Inclinometry*^*d*^^,*e*^ · Thoracolumbar spine flexion active ROM (standing) · Thoracolumbar spine extension active ROM (standing) · Thoracolumbar spine ipsilateral lateral flexion active ROM (standing) · Thoracolumbar spine contralateral lateral flexion active ROM (standing) · Hip ipsilateral internal rotation active ROM (prone position) · Hip contralateral internal rotation active ROM (prone position) *Muscle endurance time*^*d*^ · Thoracolumbar spine flexors muscles (supine position) · Thoracolumbar spine extensors muscles (supine position) · Thoracolumbar spine ipsilateral lateral flexors muscles (lateral decubitus) · Thoracolumbar spine contralateral lateral flexors muscles (lateral decubitus) · Hip ipsilateral extensors muscles (prone position) · Hip contralateral extensors muscles (prone position) · Hip ipsilateral abductors muscles (lateral decubitus) · Hip contralateral abductors muscles (lateral decubitus)	n = 102 (51 with pain) Age = 44.2 ± 8.4 Women = 75%
Choi & Chung, 2023 [29]	Republic of Korea, English	Cross-sectional	Subacromial impingement syndrome diagnosed by clinical (not described) and imaging examinations	Individuals without shoulder pain	*Radiographic imaging*: *vertebral centroid angle* · Thoracolumbar spine flexion active ROM · Thoracolumbar spine extension active ROM · Thoracolumbar spine active ROM on the sagittal plane	n = 108 (55 with pain) Age = 50.1 ± 3.8 Women = 45%
Rebelatto et al., 2023 [30]	Brazil, English	Cross-sectional	Shoulder pain (> 4 weeks) during active shoulder elevation, and with intensity ≥ 4 points in the Numerical Pain Rating Scale (0–10)	Individuals without shoulder pain	*Cervical Spine ROM device (seated)*^*d*^ · Cervical spine flexion active ROM · Cervical spine extension active ROM · Cervical spine ipsilateral lateral flexion active ROM · Cervical spine contralateral lateral flexion active ROM · Cervical spine ipsilateral rotation active ROM · Cervical spine contralateral rotation active ROM *Handheld dynamometry*^*d*^ · Cervical spine flexors muscles (supine position) · Cervical spine extensors muscles (supine position) · Cervical spine ipsilateral lateral flexors muscles (lateral decubitus) · Cervical spine contralateral lateral flexors muscles (lateral decubitus)	n = 96 (48 with pain) Age = 35.3 ± 10.5 Women = 33%

Abbreviations: ROM, range of motion.

^a^ Mean age (years) is presented with ± standard deviation.

^b^ Measures were taken using a plurimeter and a pluricompass.

^c^ Median and range data were transformed into mean ± standard deviation using Wan et al. 2014 formulas.

^d^ In the shoulder pain group, “ipsilateral” refers to the painful side, and “contralateral” to the side without pain. In the control group, sides were pair-distributed according to the hand dominance of individuals from the former group.

^e^ Measures were taken using a smartphone inclinometer application.

According to the JBI tool, none of the six included studies [5,26–30] presented a high risk of bias (S4 File). The GRADE analysis classified the quality of evidence as “very low”, considering the observational design of the included studies and the different levels of inconsistency, indirectiness, and/or imprecision (Table 2).

**Table 2:** Quality of the evidence according to the GRADE approach.

Outcomes	Authors, publication year	n	Effect estimate (95% CI)	GRADE
*ROM* ^*a*^				
Cervical spine flexion ROM (°)	Rebelatto et al., 2023 [30]	96 (48 with shoulder pain)	-4.10 [-8.54, 0.34]	⊕◯◯◯ Very low*
Cervical spine extension ROM^b^ (°)	Rebelatto et al., 2023 [30]	96 (48 with shoulder pain)	-2.10 [-6.10, 1.90]	⊕◯◯◯ Very low*
Cervical spine lateral flexion ROM: IL (°)	Rebelatto et al., 2023 [30]	96 (48 with shoulder pain)	-3.90 [-7.86, 0.06]	⊕◯◯◯ Very low*
Cervical spine lateral flexion ROM: CL (°)	Rebelatto et al., 2023 [30]	96 (48 with shoulder pain)	-3.40 [-7.31, 0.51]	⊕◯◯◯ Very low*
Cervical spine rotation ROM: IL (°)	Rebelatto et al., 2023 [30]	96 (48 with shoulder pain)	-3.30 [-7.04, 0.44]	⊕◯◯◯ Very low*
Cervical spine rotation ROM: CL (°)	Rebelatto et al., 2023 [30]	96 (48 with shoulder pain)	-3.60 [-7.33, 0.13]	⊕◯◯◯ Very low*
Thoracolumbar flexion ROM (°)	Theisen et al., 2010 [27] Hunter et al., 2020 [26] Maciel & Sousa, 2022 [5] Choi & Chung, 2023 [29]	366 (184 with shoulder pain)	-0.08 [-0.50, 0.34]	⊕◯◯◯ Very low^§^^‡^*
Thoracolumbar extension ROM (°)	Theisen et al., 2010 [27] Hunter et al., 2020 [26] Maciel & Sousa, 2022 [5] Choi & Chung, 2023 [29]	366 (184 with shoulder pain)	0.69 [-0.69, 2.07]	⊕◯◯◯ Very low^§^^‡^*
Thoracolumbar ROM: sagittal plane (°)	Meurer et al., 2004 [28] Theisen et al., 2010 [27] Hunter et al., 2020 [26] Choi & Chung, 2023 [29]	364 (183 with shoulder pain)	-0.65 [-0.86, -0.44]	⊕◯◯◯ Very low^‡^*
Thoracolumbar lateral flexion ROM: IL (°)	Maciel & Sousa, 2022 [5]	102 (51 with shoulder pain)	-3.10 [-5.46, -0.74]	⊕◯◯◯ Very low*
Thoracolumbar lateral flexion ROM: CL (°)	Maciel & Sousa, 2022 [5]	102 (51 with shoulder pain)	-5.60 [-7.91, -3.29]	⊕◯◯◯ Very low*
Thoracolumbar ROM: frontal plane (°)	Meurer et al., 2004^28^	102 (51 with shoulder pain)	-9.30 [-15.95, -2.65]	⊕◯◯◯ Very low*
Thoracolumbar ROM: transverse plane (°)	Meurer et al., 2004 [28]	102 (51 with shoulder pain)	-13.70 [-19.60, -7.80]	⊕◯◯◯ Very low*
Hip internal rotation ROM: IL (°)	Maciel & Sousa, 2022 [5]	102 (51 with shoulder pain)	-4.70 [-8.24, -1.16]	⊕◯◯◯ Very low*
Hip external rotation ROM: CL (°)	Maciel & Sousa, 2022 [5]	102 (51 with shoulder pain)	-4.20 [-7.95, -0.45]	⊕◯◯◯ Very low*
*Isometric muscular strength* ^*a*^				
Cervical spine flexors isometric strength (kgF)	Rebelatto et al., 2023 [30]	96 (48 with shoulder pain)	0.50 [-1.34, 2.34]	⊕◯◯◯ Very low*
Cervical spine extensors isometric strength^b^ (kgF)	Rebelatto et al., 2023 [30]	96 (48 with shoulder pain)	1.20 [-1.30, 3.70]	⊕◯◯◯ Very low*
Cervical spine lateral flexors isometric strength: IL (kgF)	Rebelatto et al., 2023 [30]	96 (48 with shoulder pain)	0.00 [-1.78, 1.78]	⊕◯◯◯ Very low*
Cervical spine lateral flexors isometric strength: CL (kgF)	Rebelatto et al., 2023 [30]	96 (48 with shoulder pain)	0.70 [-1.26, 2.66]	⊕◯◯◯ Very low*
*Posture*				
Static thoracic posture (°)	Meurer et al., 2004 [28] Theisen et al., 2010 [27] Hunter et al., 2020 [26]	256 (128 with shoulder pain)	0.28 [-0.06, 0.63]	⊕◯◯◯ Very low^‡^*
*Endurance* ^*a*^				
Thoracolumbar flexors endurance (s)	Maciel & Sousa, 2022 [5]	102 (51 with shoulder pain)	-16.80 [-27.75, -5.85]	⊕◯◯◯ Very low*
Thoracolumbar extensors endurance (s)	Maciel & Sousa, 2022 [5]	102 (51 with shoulder pain)	-54.70 [-76.59, -32.81]	⊕◯◯◯ Very low*
Thoracolumbar lateral flexors endurance: IL (s)	Maciel & Sousa, 2022 [5]	102 (51 with shoulder pain)	-21.00 [-33.84, -8.16]	⊕◯◯◯ Very low*
Thoracolumbar lateral flexors endurance: CL (s)	Maciel & Sousa, 2022 [5]	102 (51 with shoulder pain)	-26.80 [-40.50, -13.10]	⊕◯◯◯ Very low*
Hip extensors endurance: IL (s)	Maciel & Sousa, 2022 [5]	102 (51 with shoulder pain)	-31.80 [-47.08, -16.52]	⊕◯◯◯ Very low*
Hip extensors endurance: CL (s)	Maciel & Sousa, 2022 [5]	102 (51 with shoulder pain)	-38.10 [-58.93, -17.27]	⊕◯◯◯ Very low*
Hip abductors endurance: IL (s)	Maciel & Sousa, 2022 [5]	102 (51 with shoulder pain)	-30.70 [-45.37, -16.03]	⊕◯◯◯ Very low*
Hip abductors endurance: CL (s)	Maciel & Sousa, 2022 [5]	102 (51 with shoulder pain)	-33.10 [-47.66, -18.54]	⊕◯◯◯ Very low*
*Neuromuscular control* ^*a*,*c*^				
Neuromuscular control: IL (%)	Maciel & Sousa, 2022 [5]	102 (51 with shoulder pain)	-4.10 [-7.79, -0.41]	⊕◯◯◯ Very low*
Neuromuscular control: CL (%)	Maciel & Sousa, 2022 [5]	102 (51 with shoulder pain)	-7.50 [-11.12, -3.88]	⊕◯◯◯ Very low*

Abbreviations: CI, confidence interval; GRADE, Grading of Recommendations, Assessment, Development, and Evaluation; ROM, range of motion;°, degrees; 95%; IL, ipsilateral; CL, contralateral; kgF, kilogram-force; s, seconds; %; percentage.

^a^ In the shoulder pain group, “ipsilateral” refers to the painful side, and “contralateral” to the side without pain. In the control group, sides were pair-distributed according to the hand dominance of individuals from the former group.

^b^ Median and range data were transformed into mean ± standard deviation using Wan et al. 2014 formulas.

^c^ Values referring to the compound reach result for the test (sum of the reaches in the anterior, posteromedial, and posterolateral directions normalized by the lower limb length).

^§^ Inconsistency: statistical heterogeneity between studies (I^2^ > 50%).

^‡^ Indirectness: clinical heterogeneity between studies (different methods of assessment).

* Imprecision: sparse data (< 200 individuals in the comparison and/or 95% CI crossing the 0.5 mark in any direction).

### Cervical spine active ROM and isometric muscular strength

**Fig 2:**
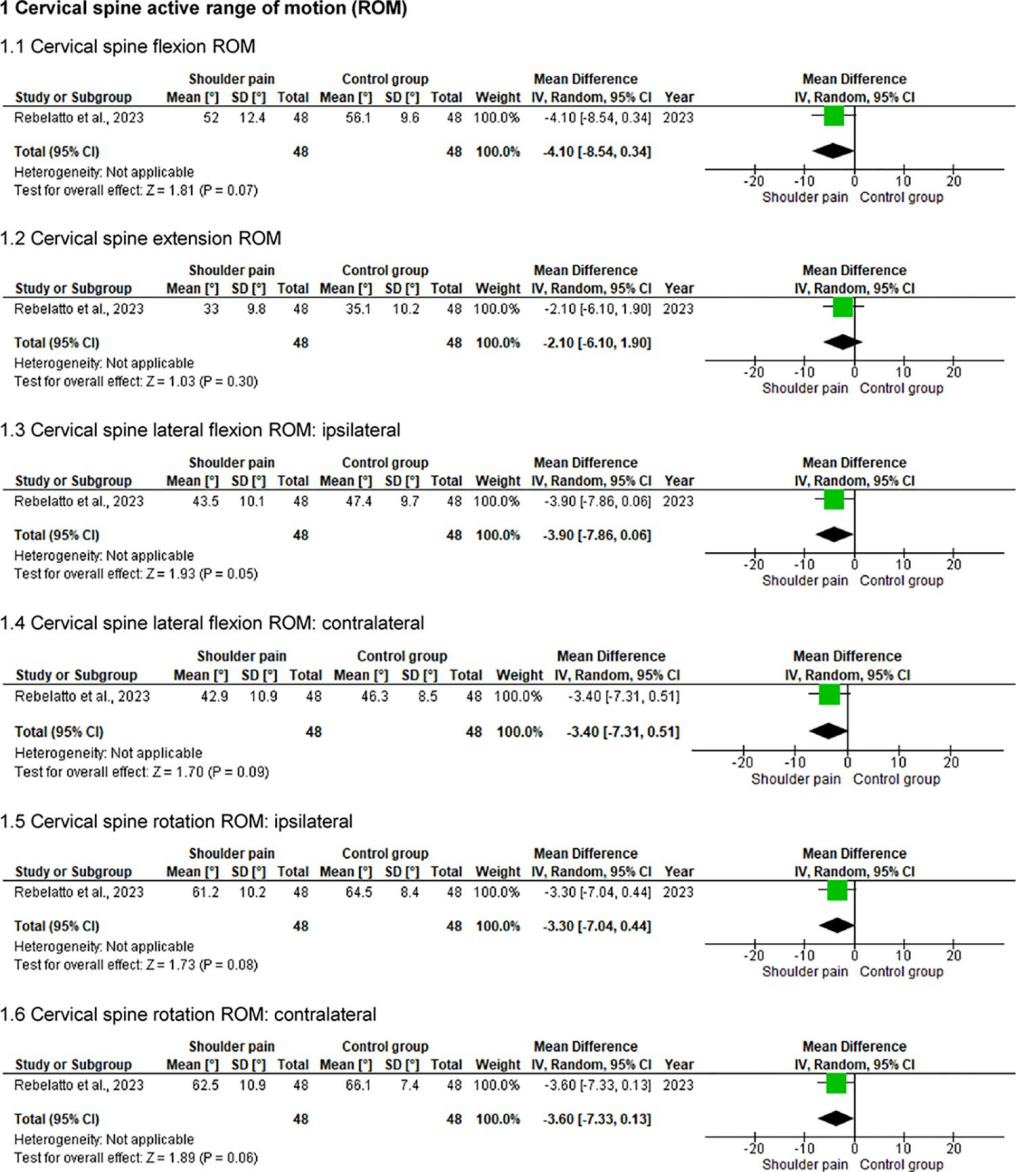
Forest plots of the cervical spine ROM in individuals with and without shoulder pain.

**Fig 3:**
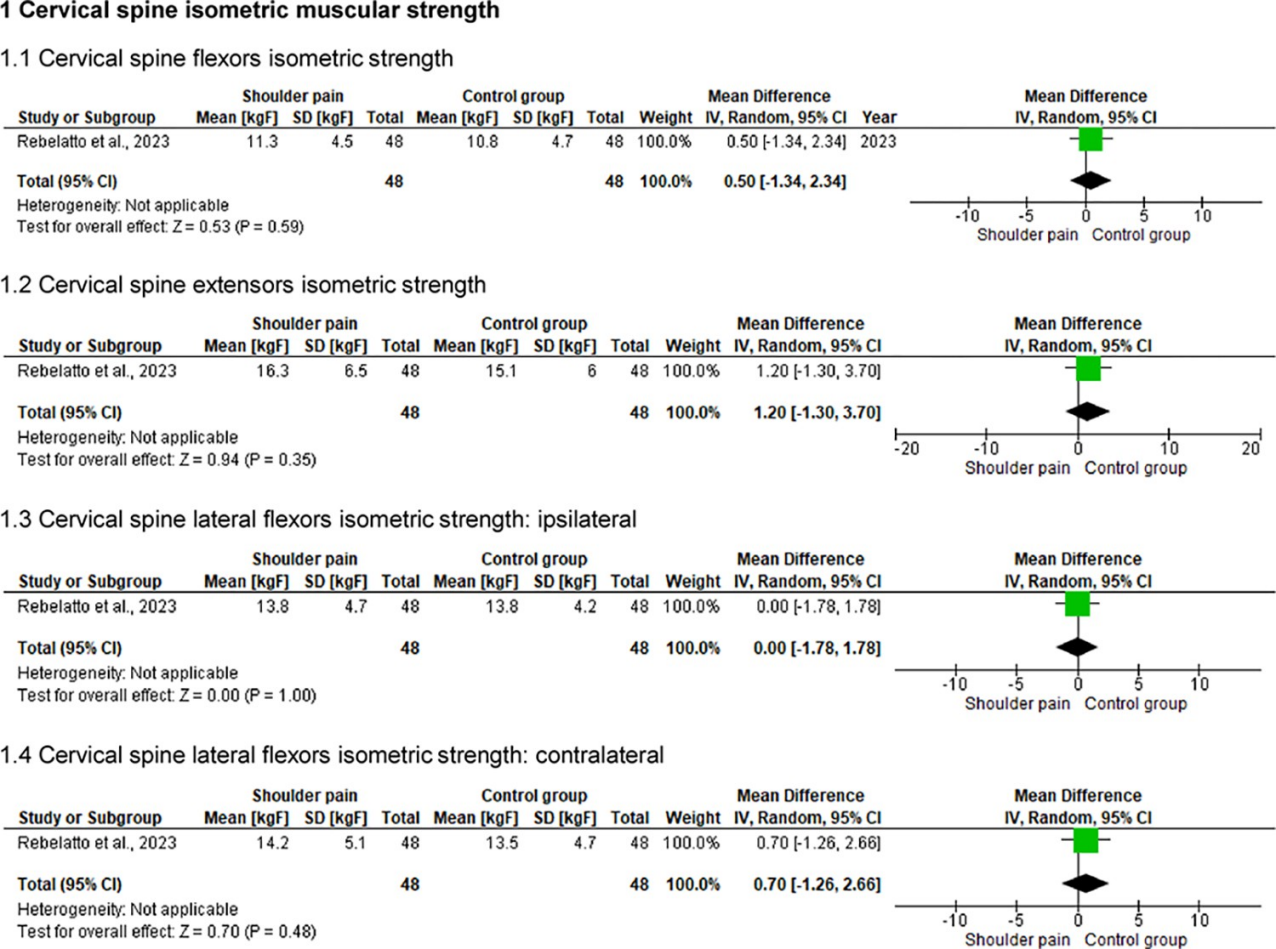
Forest plots of the cervical spine isometric strength in individuals with and without shoulder pain.

One study [30] (n = 96) compared the cervical spine active ROM (i.e., flexion, extension, lateral flexions, and rotations) and isometric muscular strength (i.e., flexors, extensors, and lateral flexors) between 48 individuals with and 48 without shoulder pain (Figs 2 and 3). For active ROM, statistically significant differences were found only for cervical spine extension (p < .05), with individuals with shoulder pain showing decreased active mobility (approximately 6°). There were no differences between the groups for isometric strength assessments (p > .05).

### Thoracic spine posture

**Fig 4:**
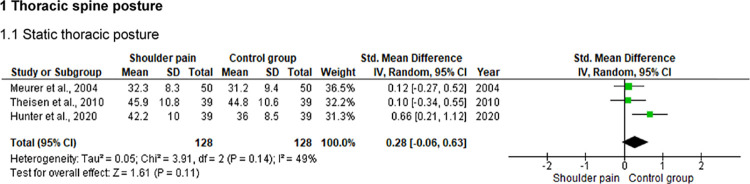
Forest plot of the thoracic spine posture in individuals with and without shoulder pain.

Three studies [26–28] compared the static thoracic spine posture (i.e., the static thoracic kyphosis) between 128 individuals with and 128 without shoulder pain using different clinical and imaging methods (Fig 4). The radiographic assessment pointed to statistically significant differences between groups (p < .01), with the shoulder pain group showing increased thoracic kyphosis compared to asymptomatic individuals (a mean difference of approximately 6°) [26]. However, measures taken using inclinometry [28] or ultrasound topometry [27] did not show significant differences between groups (p ≥ .08).

### Thoracolumbar region active ROM on the sagittal plane

**Fig 5:**
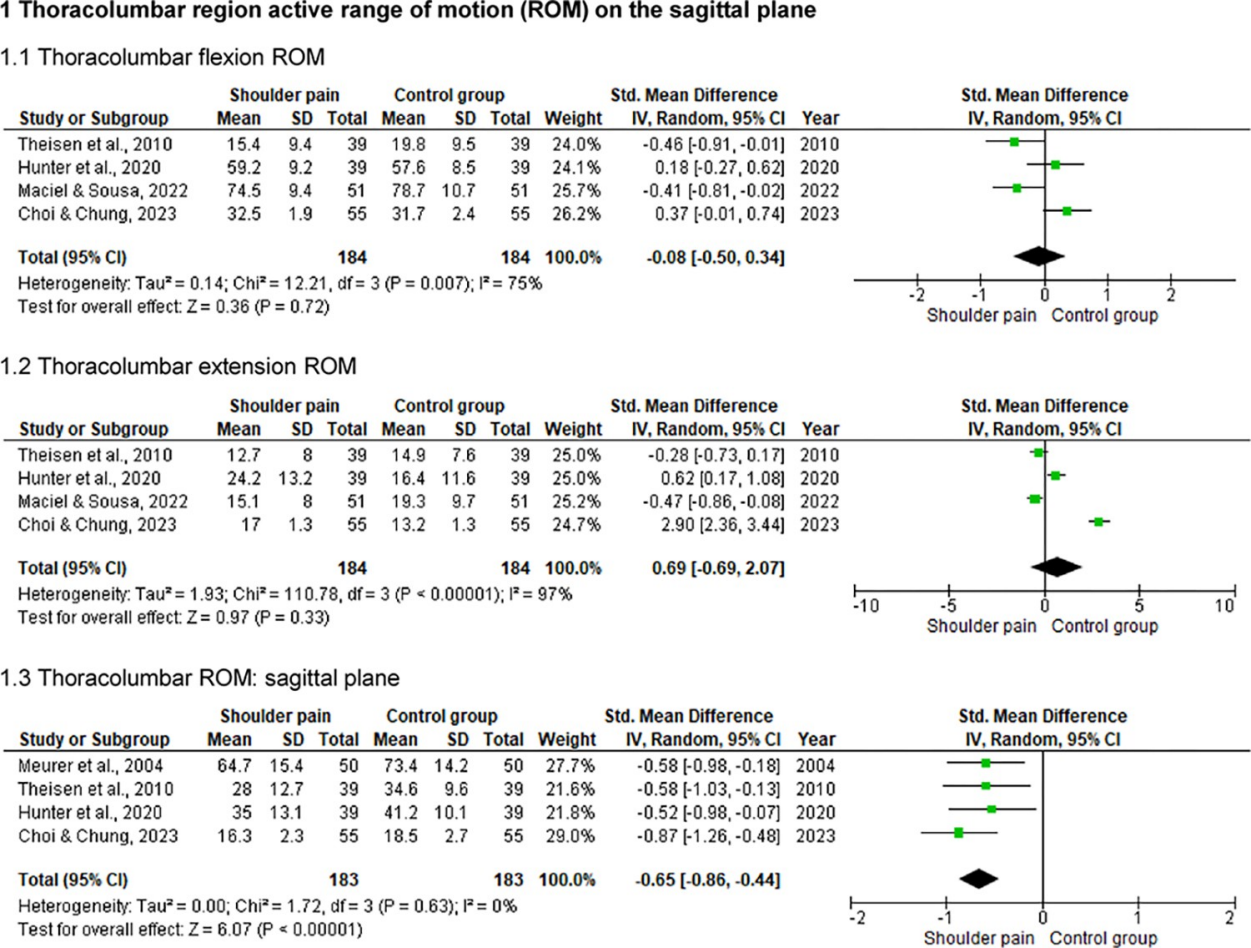
Forest plots of the thoracolumbar spine ROM on the sagittal plane in individuals with and without shoulder pain.

Five studies [5,26–29] with a pooled sample of 466 individuals (234 with shoulder pain) assessed the thoracolumbar active ROM on the sagittal plane (Fig 5). Statistically significant differences between individuals with and without shoulder pain were reported for flexion (p = .03) [5], extension (p ≤ .03) [5,26,29], and total ROM of the thoracolumbar region on the sagittal plane (p < .02) [26–29]. Symptomatic individuals presented a relatively lower total ROM on those assessments (between approximately 2° and 9°).

### Total thoracolumbar region active ROM on frontal and transverse planes

**Fig 6:**
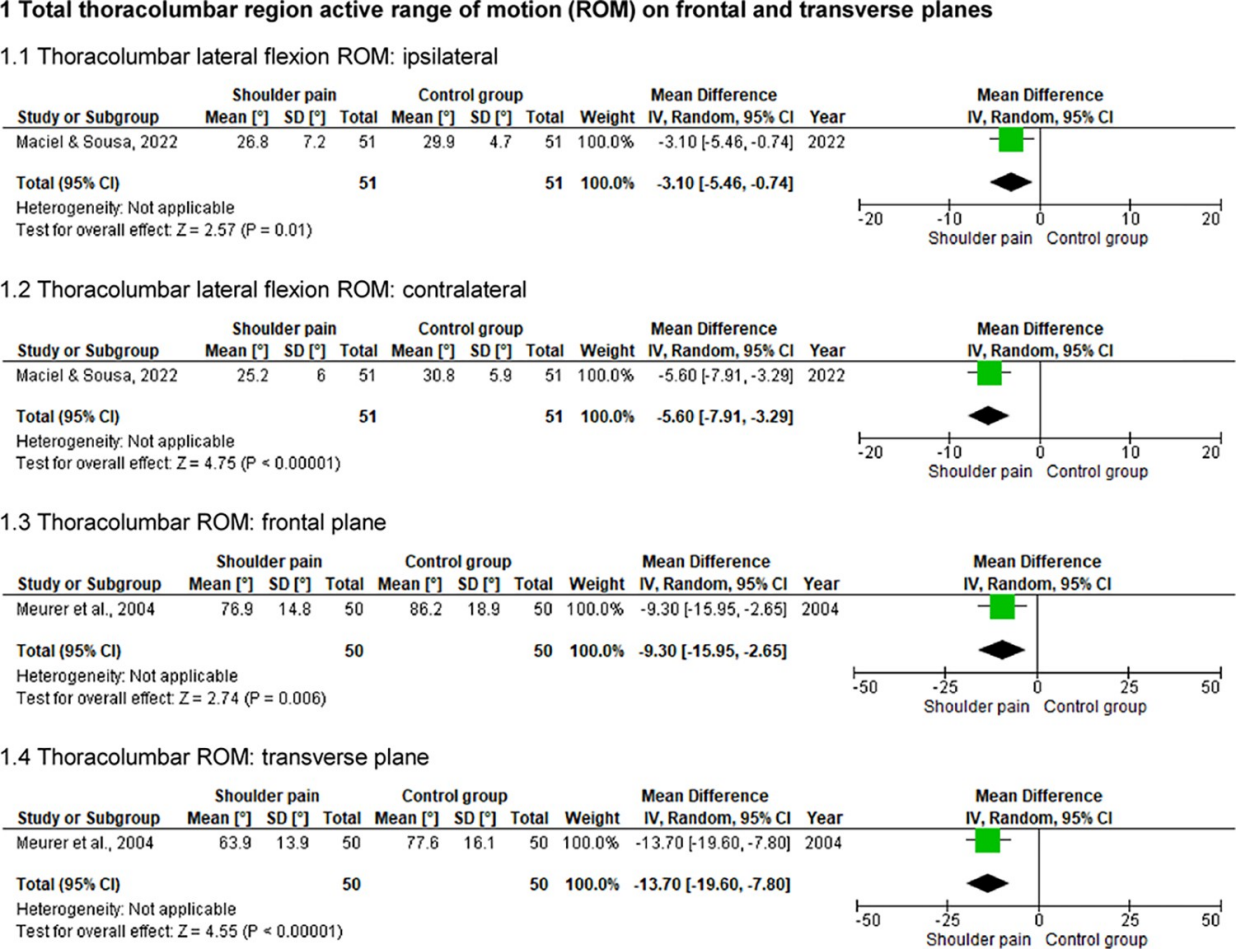
Forest plots of the thoracolumbar spine ROM on frontal and transverse planes in individuals with and without shoulder pain.

Two studies with a pooled sample of 202 individuals (101 with shoulder pain) assessed the thoracolumbar active lateral flexions [5,28] and rotations [28] ROM using an inclinometer (Fig 6). Symptomatic individuals presented a lower thoracolumbar ROM (p ≤ .02) (differences of approximately 9° and 14° on frontal and transverse planes, respectively).

### Hip internal rotation active ROM

**Fig 7:**
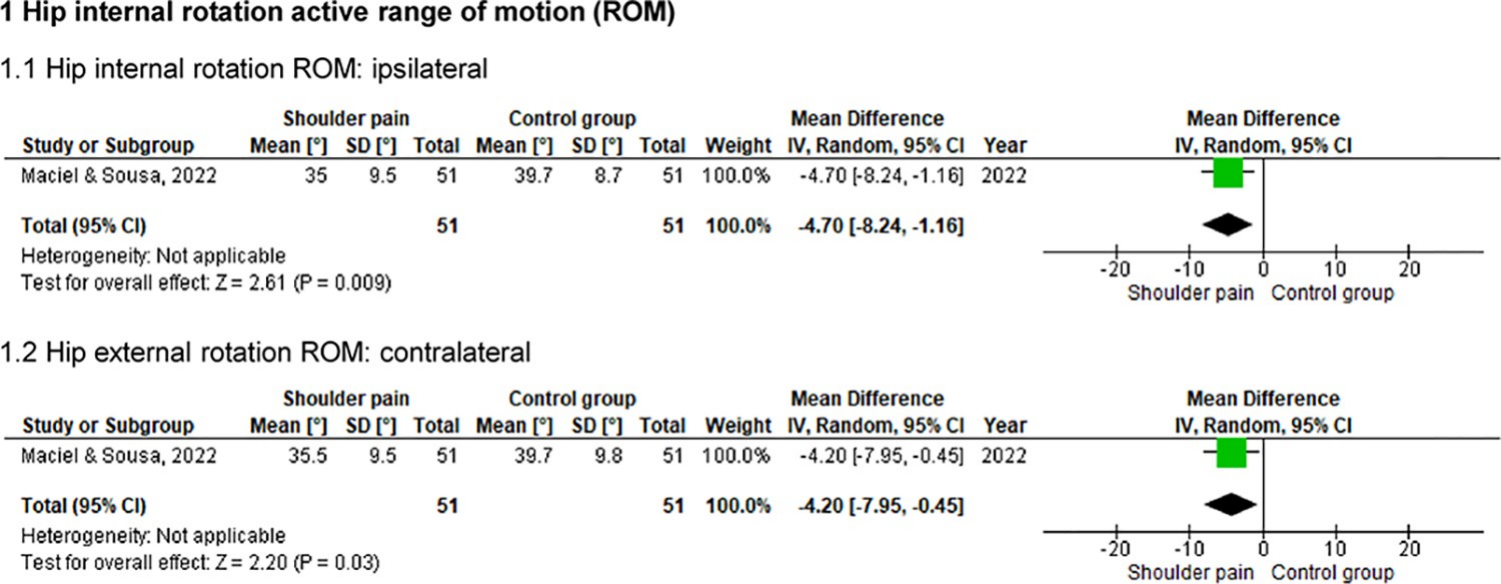
Forest plots of the hip ROM in individuals with and without shoulder pain.

One study [5] (n = 102) assessed the hip active internal rotation ROM using a smartphone inclinometer application (Fig 7). Results showed that symptomatic individuals (n = 51) presented lower hip internal rotation ROM (between approximately 4° and 5°, p ≤ .03).

### Thoracolumbar region and hip muscular endurance and neuromuscular control of the lower extremities

**Fig 8:**
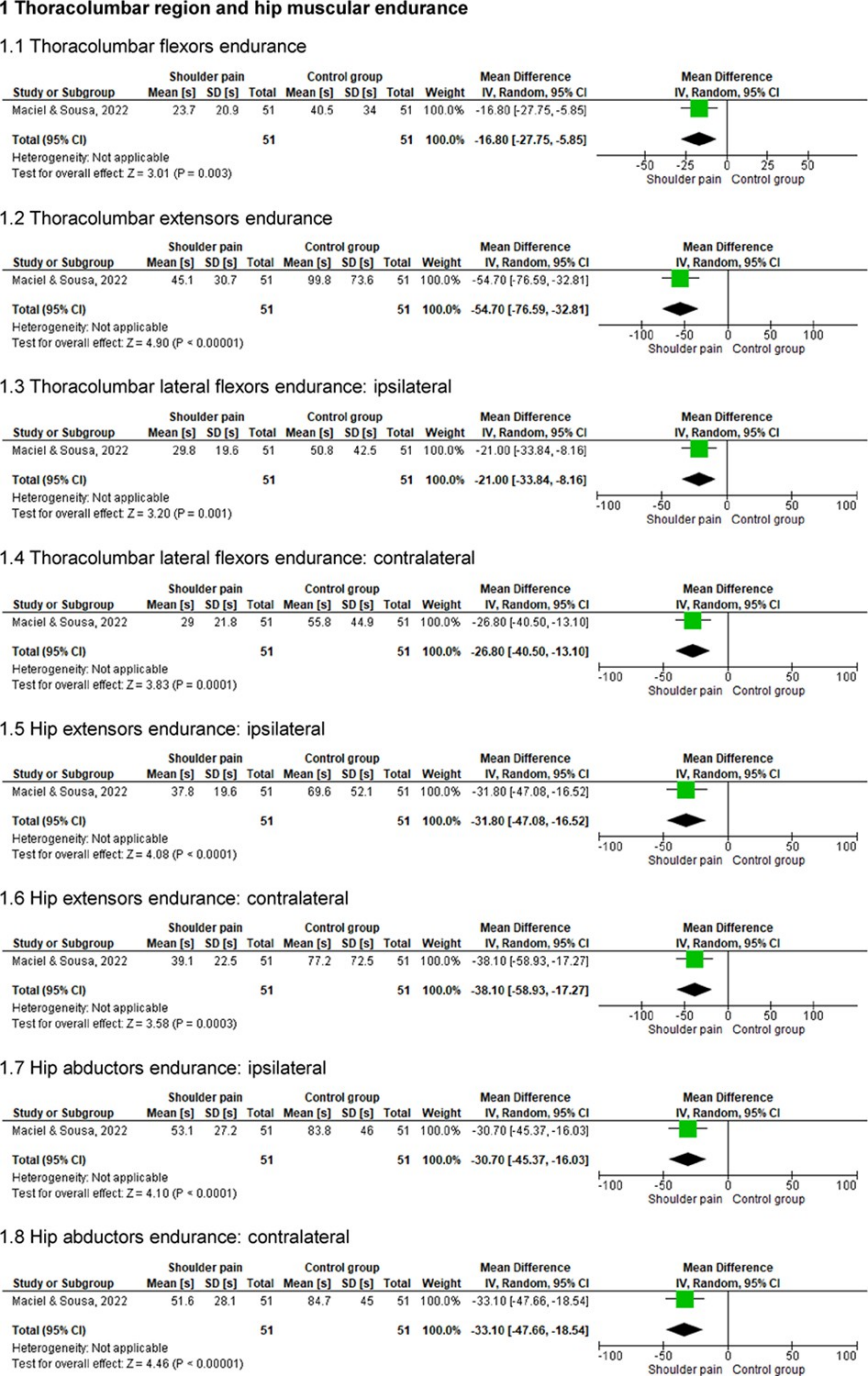
Forest plots of the thoracolumbar region and hip endurance in individuals with and without shoulder pain.

**Fig 9:**
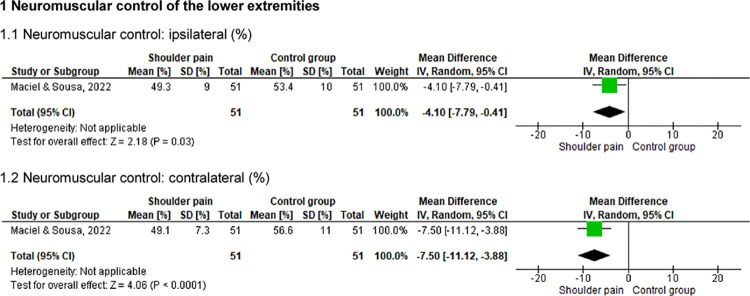
Forest plots of the neuromuscular control in individuals with and without shoulder pain.

One study [5] assessed the endurance of the thoracolumbar (flexors, extensors, and lateral flexors) and hip (extensors and abductors) muscles and the neuromuscular control of the lower extremities of 102 individuals (51 with shoulder pain) (Figs 8 and 9). This study indicated that symptomatic individuals presented lower muscular endurance in the thoracolumbar region and hip, as well as decreased neuromuscular control of the lower extremities (p ≤ .01).

## Discussion

This review synthesized the evidence about the characteristics of the kinetic chain of non-athletes with shoulder pain compared to asymptomatic individuals. Six cross-sectional studies [5,26–30] with a low risk of bias were included and very low quality evidence suggests that individuals with shoulder pain may present reduced active ROM (i.e., cervical spine, thoracolumbar region, and hip), muscular endurance (i.e., thoracolumbar region and hip), and neuromuscular control of the lower extremities. Results related to thoracic spine posture were conflicting. These findings have already been observed in previous studies in athletes of different modalities involving throwing gestures [16] but, to the authors’ knowledge, this is the first systematic review to summarize such information in the non-athletic population.

Regarding the characteristics of the cervical spine region in individuals with shoulder pain, a study [30] concluded that cervical extension ROM may be reduced in non-athletes with shoulder pain. The authors observed that for each degree decrease in mobility, individuals were 1.1 times more likely to experience shoulder pain [30]. On the other hand, no relationships were observed for the other analyses of cervical spine active ROM (in the sagittal, frontal, and transverse planes) and isometric muscular strength. Although assessments of the cervical spine appear relevant in the kinetic chain evaluation of individuals with shoulder pain [6,30], evidence about the cervical spine characteristics in this population is still lacking.

The findings of this review were controversial on the thoracic spine posture in non-athlete individuals with shoulder pain. A study showed greater thoracic kyphosis in individuals with shoulder pain compared to those without pain, as indicated by radiographic imaging [26]. However, alternative methods of assessing thoracic posture, such as inclinometry [28] and ultrasound topometry [27], did not reveal significant differences between individuals with and without shoulder pain. It is important to highlight that, although the reliability of thoracic posture assessment through radiography has been considered good-to-excellent [26,31], the authors did not presented data regarding the reliability and feasibility of assessments using inclinometry [28] and ultrasound topometry [27]. Hence, the lack of measurement properties and a standardized assessment method may contribute to the divergent results on the thoracic spine posture. Additionally, differences in diagnostic criteria and the lack of information about the characteristics of shoulder pain (e.g., duration, chronicity, and intensity) among studies[26–28] make it difficult to compare findings, even when the samples have similar symptoms and age groups.

A previous systematic review [32] also summarized inconclusive results regarding thoracic posture in individuals with shoulder pain, which resulted in moderate evidence of no statistically significant differences between groups with and without shoulder pain. Nevertheless, the posture and mobility of the thoracic spine can influence the positioning and kinematics of the scapula and, consequently, the stability and movements of the glenohumeral joint [33,34]. Accordingly, postural changes in the thoracic spine may be observed in individuals with ROM deficits in the shoulder, even if not related to the pain outcome [32].

Therefore, this review identified that individuals with shoulder pain may present reduced total active thoracolumbar region mobility, although diverging results were found when observing the isolated thoracolumbar extension movement [5,29,35]. In addition, individuals with shoulder pain may also present decreased active ROM on the hip, another central segment of the kinetic chain [5]. Outside the sporting context, the adequate connection between these segments is important for the transmission of energy needed to carry out activities that require the shoulder complex [5]. However, since the findings of this review are based on very low quality evidence, more studies are necessary to further explore the relationship between the shoulder pain and the thoracolumbar/hip regions.

The results of this review indicated that individuals with shoulder pain presented lower endurance in muscles of the thoracolumbar region (flexors, extensors, and lateral flexors) and hip (extensors and abductors) compared to asymptomatic individuals during tests that measured the time (seconds) of maintenance of isometric contraction of these muscles [5]. The assessment of muscular endurance is relevant since this outcome is related to the maintenance of body stability during functional tasks and is necessary for repetitive and/or long-lasting activities [12,36].

Regarding the neuromuscular control of the lower extremities, individuals with shoulder pain showed reduced results on the Modified Star Excursion Balance Test [5]. Impairments in neuromuscular control may be related to changes in muscular strength, coordination, balance, postural control, and/or stability and impact the function of other body segments of the kinetic chain [9,12,37]. However, the findings of this review regarding muscular endurance and neuromuscular control were based on only one study [5]. Although the methods employed by the authors of that study were robust and reliable, the quality of the evidence was rated as very low based on the GRADE approach due to the observational design and sparse data [5]. Therefore, drawing causal relationships should be avoided.

Furthermore, there are other important factors related to the kinetic chain that were not investigated by the studies included in this review, such as muscular strength and power of other corporal segments, stability, and body coordination. Additionally, some functional and physical performance tests (such as the Closed Kinetic Chain Upper Extremity Stability Test, the Upper Quarter Y Balance Test, and the Upper Limb Rotation Test) that evaluate different constructs related to physical function along the kinetic chain are options less explored in the non-athlete public [38–42].

Concurrently, some studies [35,43–48] have already investigated the effect of interventions focusing on the cervical spine and thoracic regions of individuals with shoulder pain, but the results regarding pain, function, disability, and other outcomes are still inconclusive. In asymptomatic individuals, a systematic review [49] concluded that the integration of segments of the kinetic chain during exercises focusing on the shoulder complex can increase muscle activation of the lower trapezius and serratus anterior, generate lower trapezius ratios and reduce demands on the rotator cuff. Given the divergent evidence, future studies are needed to investigate the potential benefits of interventions focusing on components of the kinetic chain in reducing pain intensity and disability in individuals with shoulder pain.

### Clinical implications and further research

The results summarized in this review indicated that individuals with shoulder pain may present alterations in segments of the kinetic chain, suggesting that the assessment of these components may be relevant for this population. However, these results are based on a small number of cross-sectional studies with small sample size, resulting in low quality of evidence. According to the GRADE approach, this study design offers low quality of evidence compared with randomized controlled trials, unless significant limitations are present. Moreover, further methodological limitations identified in the studies included and assessed using the GRADE approach suggest that the evidence presented is of very low quality. Additionally, more studies are required to enhance the quality of evidence on the topic and explore other aspects of the kinetic chain that have not yet been examined in non-athletes.

Longitudinal studies are necessary to provide clearer evidence about the relationship between the presence of shoulder pain and changes in segments of the kinetic chain. Since the cross-sectional nature of the studies included in this review does not establish cause-effect relationships, it is not yet possible to determine which biomechanical changes in the kinetic chain are associated with the development of shoulder pain in non-athletes.

Additionally, randomized clinical trials (RCTs) should be carried out to verify the effects of rehabilitation programs that include interventions focused on components of the kinetic chain in individuals with shoulder pain. The findings from the RCTs can identify the effects of interventions focused on biomechanical factors related to the kinetic chain on shoulder pain and disability. In addition, they can determine whether these factors are modifiable through physiotherapy interventions and if these modifications lead to clinical improvements (e.g., improvement in cervical extension ROM is associated with a decrease in shoulder pain).

### Strengths and limitations

To the authors’ knowledge, this is the first systematic review that gathered studies that investigated the characteristics of the kinetic chain of non-athlete individuals with shoulder pain. This review was prospectively registered on PROSPERO, followed PRISMA recommendations, employed a comprehensive search strategy, and assessed the quality of evidence using the GRADE approach. Although the included studies indicated that individuals with shoulder pain may present alterations in components of the kinetic chain compared to asymptomatic individuals, the results of this review should be interpreted with caution due to the very low quality of evidence.

## Conclusion

Very low quality evidence suggests that non-athlete individuals with shoulder pain may present reduced ROM and muscular endurance (thoracolumbar region and hip), as well as deficits in the neuromuscular control of the lower extremities. Such findings suggest that a more comprehensive assessments that consider additional factors beyond the shoulder, including segments of the kinetic chain, may be clinically important in this population.

## Supporting information

S1 File PRISMA checklist. (DOCX)

S2 File Search strategies. (DOCX)

S3 File Extraction form. (XLSX)

S4 File Methodological quality and risk of bias assessment. (DOCX)

S5 File Studies. (XLSX)
