# Supplementary material for: Thinking outside the shoulder: A systematic review and metanalysis of kinetic chain characteristics in non-athletes with shoulder pain
Source: PLoS One. 2024 Dec 9;19(12):e0314909. doi: 10.1371/journal.pone.0314909 (PMC11627437; doi:10.1371/journal.pone.0314909)
Supplement: S2 File — (DOCX) [file pone.0314909.s002.docx]

**MEDLINE/PubMed (National Library of Medicine) - 15/02/24**

|  | "shoulder"[MeSH Terms] AND 2022:2024[Date - Publication] | **2,000** |
| --- | --- | --- |
|  | "shoulder pain"[MeSH Terms] AND 2022:2024[Date - Publication] | **635** |
|  | "shoulder joint"[MeSH Terms] AND 2022:2024[Date - Publication] | **1,933** |
|  | "shoulder injuries"[MeSH Terms] AND 2022:2024[Date - Publication] | **2,302** |
|  | shoulder impingement [Title/Abstract] AND 2022:2024[Date - Publication] | **72** |
|  | subacromial impingement [Title/Abstract] AND 2022:2024[Date - Publication] | **124** |
|  | subacromial pain [Title/Abstract] AND 2022:2024[Date - Publication] | **50** |
|  | rotator cuff [Title/Abstract] AND 2022:2024[Date - Publication] | **2,916** |
|  | glenohumeral [Title/Abstract] AND 2022:2024[Date - Publication] | **1,288** |
|  |  |  |
|  | #1 OR #2 OR #3 OR #4 OR #5 OR #6 OR #7 OR #8 OR #9 | **6,564** |
|  |  |  |
|  | kinetic chain [Title/Abstract] AND 2022:2024[Date - Publication] | **174** |
|  | energy transmission [Title/Abstract] AND 2022:2024[Date - Publication] | **98** |
|  | force transmission [Title/Abstract] AND 2022:2024[Date - Publication] | **205** |
|  | ("lower extremity"[Title/Abstract] OR "lower limb"[Title/Abstract]) AND 2022:2024[Date - Publication] | **14,435** |
|  | ("ankle"[Title/Abstract] OR "foot"[Title/Abstract]) AND 2022:2024[Date - Publication] | **21,509** |
|  | knee [Title/Abstract] AND 2022:2024[Date - Publication] | **25,258** |
|  | ("hip"[Title/Abstract] OR "pelvi*"[Title/Abstract]) AND 2022:2024[Date - Publication] | **40,062** |
|  | lumbopelvic [Title/Abstract] AND 2022:2024[Date - Publication] | **262** |
|  | ("trunk"[Title/Abstract] OR "thoracolumbar"[Title/Abstract]) AND 2022:2024[Date - Publication] | **9,134** |
|  | ("torso"[Title/Abstract] OR "spine"[Title/Abstract]) AND 2022:2024[Date - Publication] | **20,578** |
|  | ("thoracic"[Title/Abstract] OR "core"[Title/Abstract]) AND 2022:2024[Date - Publication] | **79,172** |
|  | ("cervical"[Title/Abstract] OR "neck"[Title/Abstract]) AND 2022:2024[Date - Publication] | **57,262** |
|  |  |  |
|  | #11 OR #12 OR #13 OR #14 OR #15 OR #16 OR #17 OR #18 OR #19 OR #20 OR #21 OR #22 | **231,160** |
|  |  |  |
|  | range of motion [MeSH Terms] AND (2022:2024[pdat]) | **3,881** |
|  | physical fitness [MeSH Terms] AND (2022:2024[pdat]) | **2,604** |
|  | motion [MeSH Terms] AND (2022:2024[pdat]) | **4,596** |
|  | muscle strength [MeSH Terms] AND (2022:2024[pdat]) | **5,775** |
|  | motor [Title/Abstract] AND (2022:2024[pdat]) | **44,843** |
|  | mobili* [Title/Abstract] AND (2022:2024[pdat]) | **34,395** |
|  | ("performanc*"[Title/Abstract] OR "enduranc*"[Title/Abstract] OR ("muscle"[Title/Abstract] AND "strength"[Title/Abstract]) OR "stabili*"[Title/Abstract] OR "agili*"[Title/Abstract] OR "veloci*"[Title/Abstract] OR "speed*"[Title/Abstract] OR "power"[Title/Abstract] OR ("physical"[Title/Abstract] AND "activi*"[Title/Abstract]) OR ("physical"[Title/Abstract] AND "function*"[Title/Abstract]) OR ("physical"[Title/Abstract] AND "perfomanc*"[Title/Abstract]) OR ("range"[Title/Abstract] AND "motion*"[Title/Abstract]) OR ("range"[Title/Abstract] AND "movement*"[Title/Abstract]) OR "flexibili*"[Title/Abstract] OR "neuromuscular"[Title/Abstract] OR "balance"[Title/Abstract]) AND 2022:2024[Date - Publication] | **605,919** |
|  |  |  |
|  | #24 OR #25 OR #26 OR #27 OR #28 OR #29 OR #30 | **661,318** |
|  |  |  |
|  | #10 AND #23 AND #31 | **434** |
|  |  |  |
|  | ("animal*"[Title] OR "invertebrate*"[Title] OR "nonhuman*"[Title] OR "animal experiment*"[Title] OR "animal tissue*"[Title] OR "animal model*"[Title] OR "plant*"[Title] OR "fung*"[Title] OR "agricultural"[Title] OR "cadaver*"[Title] OR "arthroscop*"[Title] OR "surger*"[Title] OR "arthroplast*"[Title] OR "burn*"[Title] OR "thromboemboli*"[Title] OR "fractur*"[Title] OR "stroke"[Title] OR "herbal"[Title] OR "cancer"[Title] OR "latarjet"[Title] OR "bankart"[Title] OR "anchor"[Title] OR "sutur*"[Title] OR "concussi*"[Title] OR "brain"[Title] OR "capsulitis"[Title] OR "frozen"[Title] OR "glycem*"[Title] OR "sclero*"[Title] OR "cortical"[Title] OR "arthrit*"[Title] OR "exoskelet*"[Title] OR "blood*"[Title] OR "reliab*"[Title] OR "valid*"[Title] OR "epidemio*"[Title] OR "clinimetric"[Title] OR "prevalence"[Title] OR "incidence"[Title] OR "spinal cord"[Title] OR "wheelchair"[Title]) AND 2022:2024[Date - Publication] | **529,290** |
|  |  |  |
|  | #32 NOT #33 | **285** |

**MEDLINE/PubMed (National Library of Medicine) - 16/12/22**

|  | shoulder [MeSH Terms] | **15,277** |
| --- | --- | --- |
|  | shoulder pain [MeSH Terms] | **5,724** |
|  | shoulder joint [MeSH Terms] | **21,173** |
|  | shoulder injuries [MeSH Terms] | **21,569** |
|  | shoulder impingement [Title/Abstract] | **747** |
|  | subacromial impingement [Title/Abstract] | **940** |
|  | subacromial pain [Title/Abstract] | **8,600** |
|  | rotator cuff [Title/Abstract] | **14,785** |
|  | glenohumeral [Title/Abstract] | **7,849** |
|  |  |  |
|  | #1 OR #2 OR #3 OR #4 OR #5 OR #6 OR #7 OR #8 OR #9 | **52,572** |
|  |  |  |
|  | kinetic chain [Title/Abstract] | **919** |
|  | energy transmission [Title/Abstract] | **521** |
|  | force transmission [Title/Abstract] | **1,304** |
|  | (lower extremity [Title/Abstract]) OR (lower limb [Title/Abstract]) | **82,029** |
|  | (ankle [Title/Abstract]) OR (foot [Title/Abstract]) | **160,798** |
|  | knee [Title/Abstract] | **169,314** |
|  | (hip [Title/Abstract]) OR (pelvi* [Title/Abstract]) | **320,002** |
|  | lumbopelvic [Title/Abstract] | **1,238** |
|  | (trunk [Title/Abstract]) OR (thoracolumbar [Title/Abstract]) | **71,392** |
|  | (torso [Title/Abstract]) OR (spine [Title/Abstract]) | **150,717** |
|  | (thoracic [Title/Abstract]) OR (core [Title/Abstract]) | **513,629** |
|  | (cervical [Title/Abstract]) OR (neck [Title/Abstract]) | **473,174** |
|  |  |  |
|  | #11 OR #12 OR #13 OR #14 OR #15 OR #16 OR #17 OR #18 OR #19 OR #20 OR #21 OR #22 | **1,702,629** |
|  |  |  |
|  | range of motion [MeSH Terms] | **59,141** |
|  | physical fitness [MeSH Terms] | **35,641** |
|  | motion [MeSH Terms] | **69,960** |
|  | muscle strength [MeSH Terms] | **43,259** |
|  | motor [Title/Abstract] | **363,653** |
|  | mobili* [Title/Abstract] | **265,411** |
|  | "performanc*"[Title/Abstract] OR "enduranc*"[Title/Abstract] OR ("muscle"[Title/Abstract] AND "strength"[Title/Abstract]) OR "stabili*"[Title/Abstract] OR "agili*"[Title/Abstract] OR "veloci*"[Title/Abstract] OR "speed*"[Title/Abstract] OR "power"[Title/Abstract] OR ("physical"[Title/Abstract] AND "activi*"[Title/Abstract]) OR ("physical"[Title/Abstract] AND "function*"[Title/Abstract]) OR ("physical"[Title/Abstract] AND "perfomanc*"[Title/Abstract]) OR ("range"[Title/Abstract] AND "motion*"[Title/Abstract]) OR ("range"[Title/Abstract] AND "movement*"[Title/Abstract]) OR "flexibili*"[Title/Abstract] OR "neuromuscular"[Title/Abstract] OR "balance"[Title/Abstract] | **3,381,000** |
|  |  |  |
|  | #24 OR #25 OR #26 OR #27 OR #28 OR #29 OR #30 | **3,921,338** |
|  |  |  |
|  | #10 AND #23 AND #31 | **3,349** |
|  |  |  |
|  | "animal*"[Title] OR "invertebrate*"[Title] OR "nonhuman*"[Title] OR "animal experiment*"[Title] OR "animal tissue*"[Title] OR "animal model*"[Title] OR "plant*"[Title] OR "fung*"[Title] OR "agricultural"[Title] OR "cadaver*"[Title] OR "arthroscop*"[Title] OR "surger*"[Title] OR "arthroplast*"[Title] OR "burn*"[Title] OR "thromboemboli*"[Title] OR "fractur*"[Title] OR "stroke"[Title] OR "herbal"[Title] OR "cancer"[Title] OR "latarjet"[Title] OR "bankart"[Title] OR "anchor"[Title] OR "sutur*"[Title] OR "concussi*"[Title] OR "brain"[Title] OR "capsulitis"[Title] OR "frozen"[Title] OR "glycem*"[Title] OR "sclero*"[Title] OR "cortical"[Title] OR "arthrit*"[Title] OR "exoskelet*"[Title] OR "blood*"[Title] OR "reliab*"[Title] OR "valid*"[Title] OR "epidemio*"[Title] OR "clinimetric"[Title] OR "prevalence"[Title] OR "incidence"[Title] OR "spinal cord"[Title] OR "wheelchair"[Title] | **4,172,867** |
|  |  |  |
|  | #32 NOT #33 | **2,534** |

**EMBASE (Elsevier) - 15/02/24**

|  | shoulder*:ti,ab AND [2022-2024]/py | **14,824** |
| --- | --- | --- |
|  | ‘shoulder pain’:ti,ab AND [2022-2024]/py | **1,711** |
|  | ‘shoulder joint’:ti,ab AND [2022-2024]/py | **740** |
|  | ‘shoulder injur*’:ti,ab AND [2022-2024]/py | **331** |
|  | ‘shoulder impingement’:ti,ab AND [2022-2024]/py | **72** |
|  | ‘subacromial impingement’:ti,ab AND [2022-2024]/py | **133** |
|  | ‘subacromial pain’:ti,ab AND [2022-2024]/py | **56** |
|  | ‘rotator cuff’:ti,ab AND [2022-2024]/py | **3,002** |
|  | glenohumeral:ti,ab AND [2022-2024]/py | **1,351** |
|  |  |  |
|  | #1 OR #2 OR #3 OR #4 OR #5 OR #6 OR #7 OR #8 OR #9 | **15,838** |
|  |  |  |
|  | ‘kinetic chain’:ti,ab AND [2022-2024]/py | **167** |
|  | ‘energy transmission’:ti,ab AND [2022-2024]/py | **83** |
|  | ‘force transmission’:ti,ab AND [2022-2024]/py | **208** |
|  | (‘lower extremit*’:ti,ab OR ‘lower limb*’:ti,ab) AND [2022-2024]/py | **18,762** |
|  | (ankle:ti,ab OR foot:ti,ab) AND [2022-2024]/py | **25,716** |
|  | knee:ti,ab AND [2022-2024]/py | **28,777** |
|  | (hip:ti,ab OR pelvi*:ti,ab) AND [2022-2024]/py | **53,984** |
|  | lumbopelvic:ti,ab AND [2022-2024]/py | **285** |
|  | (trunk:ti,ab OR thoracolumbar:ti,ab) AND [2022-2024]/py | **11,308** |
|  | (torso:ti,ab OR spine:ti,ab) AND [2022-2024]/py | **24,940** |
|  | (thoracic:ti,ab OR core:ti,ab) AND [2022-2024]/py | **91,147** |
|  | (cervical:ti,ab OR neck:ti,ab) AND [2022-2024]/py | **73,522** |
|  |  |  |
|  | #11 OR #12 OR #13 OR #14 OR #15 OR #16 OR #17 OR #18 OR #19 OR #20 OR #21 OR #22 | **282,258** |
|  |  |  |
|  | ‘range of motion’:ti,ab AND [2022-2024]/py | **8,684** |
|  | ‘physical fitness’:ti,ab AND [2022-2024]/py | **2,454** |
|  | motion:ti,ab AND [2022-2024]/py | **34,270** |
|  | ‘muscle strength’:ti,ab AND [2022-2024]/py | **7,251** |
|  | motor:ti,ab AND [2022-2024]/py | **56,171** |
|  | mobili*:ti,ab AND [2022-2024]/py | **37,545** |
|  | (performanc*:ti,ab OR enduranc*:ti,ab OR ((muscle NEAR/5 strength):ti,ab) OR stabili*:ti,ab OR agili*:ti,ab OR velocit*:ti,ab OR speed*:ti,ab OR power:ti,ab OR ((physical NEAR/5 activity):ti,ab) OR ((physical NEAR/5 function):ti,ab) OR ((physical NEAR/5 performanc*):ti,ab) OR ((range NEAR/5 motion):ti,ab) OR ((range NEAR/5 movement*):ti,ab) OR flexibili*:ti,ab OR neuromuscular:ti,ab OR balance:ti,ab) AND [2022-2024]/py | **613,480** |
|  |  |  |
|  | #24 OR #25 OR #26 OR #27 OR #28 OR #29 OR #30 | **692,550** |
|  |  |  |
|  | #10 AND #23 AND #31 | **1,582** |
|  |  |  |
|  | animal*:ti OR invertebrate*:ti OR nonhuman*:ti OR 'animal experiment*':ti OR 'animal tissue*':ti OR 'animal model*':ti OR plant*:ti OR fung*:ti OR agricultural:ti OR cadaver*:ti OR arthroscop*:ti OR surger*:ti OR arthroplast*:ti OR burn*:ti OR thromboemboli*:ti OR fractur*:ti OR stroke:ti OR herbal:ti OR cancer:ti OR latarjet:ti OR bankart:ti OR anchor:ti OR sutur*:ti OR concussi*:ti OR brain:ti OR capsulitis:ti OR frozen:ti OR glycem*:ti OR sclero*:ti OR cortical:ti OR arthrit*:ti OR exoskelet*:ti OR blood*:ti OR reliab*:ti OR valid*:ti OR epidemio*:ti OR clinimetric:ti OR prevalence:ti OR incidence:ti OR 'spinal cord':ti OR wheelchair:ti | **5,797,742** |
|  |  |  |
|  | #32 NOT #33 | **1,132** |

**EMBASE (Elsevier) - 16/12/22**

|  | shoulder*:ti,ab | **198,068** |
| --- | --- | --- |
|  | ‘shoulder pain’:ti,ab | **10,938** |
|  | ‘shoulder joint’:ti,ab | **5,444** |
|  | ‘shoulder injur*’:ti,ab | **1,676** |
|  | ‘shoulder impingement’:ti,ab | **787** |
|  | ‘subacromial impingement’:ti,ab | **1,079** |
|  | ‘subacromial pain’:ti,ab | **227** |
|  | ‘rotator cuff’:ti,ab | **16,945** |
|  | glenohumeral:ti,ab | **8,891** |
|  |  |  |
|  | #1 OR #2 OR #3 OR #4 OR #5 OR #6 OR #7 OR #8 OR #9 | **114,317** |
|  |  |  |
|  | ‘kinetic chain’:ti,ab | **1,000** |
|  | ‘energy transmission’:ti,ab | **632** |
|  | ‘force transmission’:ti,ab | **1,507** |
|  | ‘lower extremit*’:ti,ab OR ‘lower limb*’:ti,ab | **116,009** |
|  | ankle:ti,ab OR foot:ti,ab | **209,467** |
|  | knee:ti,ab | **217,481** |
|  | hip:ti,ab OR pelvi*:ti,ab | **456,007** |
|  | lumbopelvic:ti,ab | **1,498** |
|  | trunk:ti,ab OR thoracolumbar:ti,ab | **97,472** |
|  | torso:ti,ab OR spine:ti,ab | **199,048** |
|  | thoracic:ti,ab OR core:ti,ab | **649,852** |
|  | cervical:ti,ab OR neck:ti,ab | **645,548** |
|  |  |  |
|  | #11 OR #12 OR #13 OR #14 OR #15 OR #16 OR #17 OR #18 OR #19 OR #20 OR #21 OR #22 | **2,248,861** |
|  |  |  |
|  | ‘range of motion’:ti,ab | **50,836** |
|  | ‘physical fitness’:ti,ab | **14,751** |
|  | motion:ti,ab | **255,605** |
|  | ‘muscle strength’:ti,ab | **38,610** |
|  | motor:ti,ab | **485,853** |
|  | mobili*:ti,ab | **321,912** |
|  | performanc*:ti,ab OR enduranc*:ti,ab OR ((muscle NEAR/5 strength):ti,ab) OR stabili*:ti,ab OR agili*:ti,ab OR velocit*:ti,ab OR speed*:ti,ab OR power:ti,ab OR ((physical NEAR/5 activity):ti,ab) OR ((physical NEAR/5 function):ti,ab) OR ((physical NEAR/5 performanc*):ti,ab) OR ((range NEAR/5 motion):ti,ab) OR ((range NEAR/5 movement*):ti,ab) OR flexibili*:ti,ab OR neuromuscular:ti,ab OR balance:ti,ab | **3,827,551** |
|  |  |  |
|  | #24 OR #25 OR #26 OR #27 OR #28 OR #29 OR #30 | **4,562,936** |
|  |  |  |
|  | #10 AND #23 AND #31 | **10,853** |
|  |  |  |
|  | animal*:ti OR invertebrate*:ti OR nonhuman*:ti OR 'animal experiment*':ti OR 'animal tissue*':ti OR 'animal model*':ti OR plant*:ti OR fung*:ti OR agricultural:ti OR cadaver*:ti OR arthroscop*:ti OR surger*:ti OR arthroplast*:ti OR burn*:ti OR thromboemboli*:ti OR fractur*:ti OR stroke:ti OR herbal:ti OR cancer:ti OR latarjet:ti OR bankart:ti OR anchor:ti OR sutur*:ti OR concussi*:ti OR brain:ti OR capsulitis:ti OR frozen:ti OR glycem*:ti OR sclero*:ti OR cortical:ti OR arthrit*:ti OR exoskelet*:ti OR blood*:ti OR reliab*:ti OR valid*:ti OR epidemio*:ti OR clinimetric:ti OR prevalence:ti OR incidence:ti OR 'spinal cord':ti OR wheelchair:ti | **5,434,998** |
|  |  |  |
|  | #32 NOT #33 | **8,406** |

**Web of Science (Clarivate) - 15/02/24 (2022-2024)**

|  | TI=(shoulder*) OR AB=(shoulder*) | **6,427** |
| --- | --- | --- |
|  | TI=(shoulder pain) OR AB=(shoulder pain) | **1,848** |
|  | TI=(shoulder joint) OR AB=(shoulder joint) | **1,295** |
|  | TI=(shoulder injur*) OR AB=(shoulder injur*) | **988** |
|  | TI=(shoulder impingement) OR AB=(shoulder impingement) | **118** |
|  | TI=(subacromial impingement) OR AB=(subacromial impingement) | **57** |
|  | TI=(subacromial pain) OR AB=(subacromial pain) | **107** |
|  | TI=(rotator cuff) OR AB=(rotator cuff) | **1,127** |
|  | TI=(glenohumeral) OR AB=(glenohumeral) | **477** |
|  |  |  |
|  | #1 OR #2 OR #3 OR #4 OR #5 OR #6 OR #7 OR #8 OR #9 | **6,845** |
|  |  |  |
|  | TI=(kinetic chain) OR AB=(kinetic chain) | **895** |
|  | TI=(energy transmission) OR AB=(energy transmission) | **11,728** |
|  | TI=(force transmission) OR AB=(force transmission) | **2,673** |
|  | TI=(lower extremit*) OR TI=(lower limb*) OR AB=(lower extremit*) OR AB=(lower limb*) | **11,645** |
|  | TI=(ankle) OR TI=(foot) OR AB=(ankle) OR AB=(foot) | **13,454** |
|  | TI=(knee) OR AB=(knee) | **12,504** |
|  | TI=(hip) OR TI=(pelvi*) OR AB=(hip) OR AB=(pelvi*) | **19,120** |
|  | TI=(lumbopelvic) OR AB=(lumbopelvic) | **97** |
|  | TI=(trunk) OR TI=(thoracolumbar) OR AB=(trunk) OR AB=(thoracolumbar) | **5,056** |
|  | TI=(torso) OR TI=(spine) OR AB=(torso) OR AB=(spine) | **9,352** |
|  | TI=(thoracic) OR TI=(core) OR AB=(thoracic) OR AB=(core) | **77,015** |
|  | TI=(cervical) OR TI=(neck) OR AB=(cervical) OR AB=(neck) | **28,347** |
|  |  |  |
|  | #11 OR #12 OR #13 OR #14 OR #15 OR #16 OR #17 OR #18 OR #19 OR #20 OR #21 OR #22 | **172,448** |
|  |  |  |
|  | TI=(range of motion) OR AB=(range of motion) | **9,195** |
|  | TI=(physical fitness) OR AB=(physical fitness) | **2,452** |
|  | TI=(motion) OR AB=(motion) | **45,156** |
|  | TI=(muscle strength) OR AB=(muscle strength) | **5,063** |
|  | TI=(motor) OR AB=(motor) | **29,036** |
|  | TI=(mobili*) OR AB=(mobili*) | **37,299** |
|  | (((((((((((((((((((((((((((((((TI=(performanc*)) OR AB=(performanc*)) OR TI=(enduranc*)) OR AB=(enduranc*)) OR TI=(muscle AND strength)) OR AB=(muscle AND strength)) OR TI=(stabili*)) OR AB=(stabili*)) OR TI=(agili*)) OR AB=(agili*)) OR TI=(velocit*)) OR AB=(velocit*)) OR TI=(speed*)) OR AB=(speed*)) OR TI=(power)) OR AB=(power)) OR TI=(physical AND activity)) OR AB=(physical AND activity)) OR TI=(physical AND function)) OR AB=(physical AND function)) OR TI=(physical AND performanc*)) OR AB=(physical AND performanc*)) OR TI=(range AND motion)) OR AB=(range AND motion)) OR TI=(range AND movement*)) OR AB=(range AND movement*)) OR TI=(flexibili*)) OR AB=(flexibili*)) OR TI=(neuromuscular)) OR AB=(neuromuscular)) OR TI=(balance)) OR AB=(balance) | **854,636** |
|  |  |  |
|  | #24 OR #25 OR #26 OR #27 OR #28 OR #29 OR #30 | **910,941** |
|  |  |  |
|  | #10 AND #23 AND #31 | **759** |
|  |  |  |
|  | TI=(animal*) OR TI=(invertebrate*) OR TI=(nonhuman*) OR TI=(animal experiment*) OR TI=(animal tissue*) OR TI=(animal model*) OR TI=(plant*) OR TI=(fung*) OR TI=(agricultural) OR TI=(cadaver*) OR TI=(arthroscop*) OR TI=(surger*) OR TI=(arthroplast*) OR TI=(burn*) OR TI=(thromboemboli*) OR TI=(fractur*) OR TI=(stroke) OR TI=(herbal) OR TI=(cancer) OR TI=(latarjet) OR TI=(bankart) OR TI=(anchor) OR TI=(sutur*) OR TI=(concussi*) OR TI=(brain) OR TI=(capsulitis) OR TI=(frozen) OR TI=(glycem*) OR TI=(sclero*) OR TI=(cortical) OR TI=(arthrit*) OR TI=(exoskelet*) OR TI=(blood*) OR TI=(reliab*) OR TI=(valid*) OR TI=(epidemio*) OR TI=(clinimetric) OR TI=(prevalence) OR TI=(incidence) OR TI=('spinal cord') OR TI=(wheelchair) | **6,319,797** |
|  |  |  |
|  | #32 NOT #33 | **567** |

**Web of Science (Clarivate) - 16/12/22**

|  | TI=(shoulder*) OR AB=(shoulder*) | **91,693** |
| --- | --- | --- |
|  | TI=(shoulder pain) OR AB=(shoulder pain) | **20,050** |
|  | TI=(shoulder joint) OR AB=(shoulder joint) | **15,728** |
|  | TI=(shoulder injur*) OR AB=(shoulder injur*) | **11,344** |
|  | TI=(shoulder impingement) OR AB=(shoulder impingement) | **2,172** |
|  | TI=(subacromial impingement) OR AB=(subacromial impingement) | **1,142** |
|  | TI=(subacromial pain) OR AB=(subacromial pain) | **1,343** |
|  | TI=(rotator cuff) OR AB=(rotator cuff) | **12,422** |
|  | TI=(glenohumeral) OR AB=(glenohumeral) | **6,385** |
|  |  |  |
|  | #1 OR #2 OR #3 OR #4 OR #5 OR #6 OR #7 OR #8 OR #9 | **96,888** |
|  |  |  |
|  | TI=(kinetic chain) OR AB=(kinetic chain) | **18,966** |
|  | TI=(energy transmission) OR AB=(energy transmission) | **149,470** |
|  | TI=(force transmission) OR AB=(force transmission) | **39,754** |
|  | TI=(lower extremit*) OR TI=(lower limb*) OR AB=(lower extremit*) OR AB=(lower limb*) | **143,384** |
|  | TI=(ankle) OR TI=(foot) OR AB=(ankle) OR AB=(foot) | **207,096** |
|  | TI=(knee) OR AB=(knee) | **172,808** |
|  | TI=(hip) OR TI=(pelvi*) OR AB=(hip) OR AB=(pelvi*) | **299,498** |
|  | TI=(lumbopelvic) OR AB=(lumbopelvic) | **940** |
|  | TI=(trunk) OR TI=(thoracolumbar) OR AB=(trunk) OR AB=(thoracolumbar) | **80,983** |
|  | TI=(torso) OR TI=(spine) OR AB=(torso) OR AB=(spine) | **148,756** |
|  | TI=(thoracic) OR TI=(core) OR AB=(thoracic) OR AB=(core) | **1,125,881** |
|  | TI=(cervical) OR TI=(neck) OR AB=(cervical) OR AB=(neck) | **445,200** |
|  |  |  |
|  | #11 OR #12 OR #13 OR #14 OR #15 OR #16 OR #17 OR #18 OR #19 OR #20 OR #21 OR #22 | **2,575,348** |
|  |  |  |
|  | TI=(range of motion) OR AB=(range of motion) | **122,058** |
|  | TI=(physical fitness) OR AB=(physical fitness) | **28,289** |
|  | TI=(motion) OR AB=(motion) | **804,256** |
|  | TI=(muscle strength) OR AB=(muscle strength) | **52,405** |
|  | TI=(motor) OR AB=(motor) | **514,283** |
|  | TI=(mobili*) OR AB=(mobili*) | **559,038** |
|  | (((((((((((((((((((((((((((((((TI=(performanc*)) OR AB=(performanc*)) OR TI=(enduranc*)) OR AB=(enduranc*)) OR TI=(muscle AND strength)) OR AB=(muscle AND strength)) OR TI=(stabili*)) OR AB=(stabili*)) OR TI=(agili*)) OR AB=(agili*)) OR TI=(velocit*)) OR AB=(velocit*)) OR TI=(speed*)) OR AB=(speed*)) OR TI=(power)) OR AB=(power)) OR TI=(physical AND activity)) OR AB=(physical AND activity)) OR TI=(physical AND function)) OR AB=(physical AND function)) OR TI=(physical AND performanc*)) OR AB=(physical AND performanc*)) OR TI=(range AND motion)) OR AB=(range AND motion)) OR TI=(range AND movement*)) OR AB=(range AND movement*)) OR TI=(flexibili*)) OR AB=(flexibili*)) OR TI=(neuromuscular)) OR AB=(neuromuscular)) OR TI=(balance)) OR AB=(balance) | **11,026,582** |
|  |  |  |
|  | #24 OR #25 OR #26 OR #27 OR #28 OR #29 OR #30 | **12,152,165** |
|  |  |  |
|  | #10 AND #23 AND #31 | **9,195** |
|  |  |  |
|  | TI=(animal*) OR TI=(invertebrate*) OR TI=(nonhuman*) OR TI=(animal experiment*) OR TI=(animal tissue*) OR TI=(animal model*) OR TI=(plant*) OR TI=(fung*) OR TI=(agricultural) OR TI=(cadaver*) OR TI=(arthroscop*) OR TI=(surger*) OR TI=(arthroplast*) OR TI=(burn*) OR TI=(thromboemboli*) OR TI=(fractur*) OR TI=(stroke) OR TI=(herbal) OR TI=(cancer) OR TI=(latarjet) OR TI=(bankart) OR TI=(anchor) OR TI=(sutur*) OR TI=(concussi*) OR TI=(brain) OR TI=(capsulitis) OR TI=(frozen) OR TI=(glycem*) OR TI=(sclero*) OR TI=(cortical) OR TI=(arthrit*) OR TI=(exoskelet*) OR TI=(blood*) OR TI=(reliab*) OR TI=(valid*) OR TI=(epidemio*) OR TI=(clinimetric) OR TI=(prevalence) OR TI=(incidence) OR TI=('spinal cord') OR TI=(wheelchair) | **5,946,413** |
|  |  |  |
|  | #32 NOT #33 | **7,222** |

**Scopus (Elsevier) - 15/02/24**

|  | TITLE-ABS("shoulder*") AND PUBYEAR > 2021 AND PUBYEAR < 2025 | **17,449** |
| --- | --- | --- |
|  | TITLE-ABS(“shoulder pain”) AND PUBYEAR > 2021 AND PUBYEAR < 2025 | **1,604** |
|  | TITLE-ABS(“shoulder joint”) AND PUBYEAR > 2021 AND PUBYEAR < 2025 | **1,016** |
|  | TITLE-ABS(“shoulder injur*”) AND PUBYEAR > 2021 AND PUBYEAR < 2025 | **356** |
|  | TITLE-ABS(“shoulder impingement”) AND PUBYEAR > 2021 AND PUBYEAR < 2025 | **70** |
|  | TITLE-ABS(“subacromial impingement”) AND PUBYEAR > 2021 AND PUBYEAR < 2025 | **127** |
|  | TITLE-ABS(“subacromial pain”) AND PUBYEAR > 2021 AND PUBYEAR < 2025 | **59** |
|  | TITLE-ABS(“rotator cuff”) AND PUBYEAR > 2021 AND PUBYEAR < 2025 | **2,886** |
|  | TITLE-ABS(“glenohumeral”) AND PUBYEAR > 2021 AND PUBYEAR < 2025 | **1,314** |
|  |  |  |
|  | #1 OR #2 OR #3 OR #4 OR #5 OR #6 OR #7 OR #8 OR #9 | **18,484** |
|  |  |  |
|  | TITLE-ABS(“kinetic chain”) AND PUBYEAR > 2021 AND PUBYEAR < 2025 | **200** |
|  | TITLE-ABS(“energy transmission”) AND PUBYEAR > 2021 AND PUBYEAR < 2025 | **1,207** |
|  | TITLE-ABS(“force transmission”) AND PUBYEAR > 2021 AND PUBYEAR < 2025 | **633** |
|  | (TITLE-ABS(“lower extremit*”) OR TITLE-ABS(“lower limb*”)) AND PUBYEAR > 2021 AND PUBYEAR < 2025 | **22,127** |
|  | (TITLE-ABS(“ankle”) OR TITLE-ABS(“foot”)) AND PUBYEAR > 2021 AND PUBYEAR < 2025 | **33,781** |
|  | TITLE-ABS(“knee”) AND PUBYEAR > 2021 AND PUBYEAR < 2025 | **30,055** |
|  | (TITLE-ABS(“hip”) OR TITLE-ABS(“pelvi*”)) AND PUBYEAR > 2021 AND PUBYEAR < 2025 | **46,221** |
|  | TITLE-ABS(“lumbopelvic”) AND PUBYEAR > 2021 AND PUBYEAR < 2025 | **250** |
|  | (TITLE-ABS(“trunk”) OR TITLE-ABS(“thoracolumbar”)) AND PUBYEAR > 2021 AND PUBYEAR < 2025 | **13,325** |
|  | (TITLE-ABS(“torso”) OR TITLE-ABS(“spine”)) AND PUBYEAR > 2021 AND PUBYEAR < 2025 | **23,937** |
|  | (TITLE-ABS(“thoracic”) OR TITLE-ABS(“core”)) AND PUBYEAR > 2021 AND PUBYEAR < 2025 | **208,370** |
|  | (TITLE-ABS(“cervical”) OR TITLE-ABS(“neck”)) AND PUBYEAR > 2021 AND PUBYEAR < 2025 | **64,955** |
|  |  |  |
|  | #11 OR #12 OR #13 OR #14 OR #15 OR #16 OR #17 OR #18 OR #19 OR #20 OR #21 OR #22 | **400,928** |
|  |  |  |
|  | TITLE-ABS(“range of motion”) AND PUBYEAR > 2021 AND PUBYEAR < 2025 | **9,395** |
|  | TITLE-ABS(“physical fitness”) AND PUBYEAR > 2021 AND PUBYEAR < 2025 | **3,723** |
|  | TITLE-ABS(“motion”) AND PUBYEAR > 2021 AND PUBYEAR < 2025 | **127,669** |
|  | TITLE-ABS(“muscle strength”) AND PUBYEAR > 2021 AND PUBYEAR < 2025 | **6,951** |
|  | TITLE-ABS(“motor”) AND PUBYEAR > 2021 AND PUBYEAR < 2025 | **85,096** |
|  | TITLE-ABS(“mobili*”) AND PUBYEAR > 2021 AND PUBYEAR < 2025 | **101,647** |
|  | (TITLE-ABS ( performanc* OR enduranc* OR ( muscle W/ 5 strength ) OR stabili* OR agili* OR velocit* OR speed* OR power OR ( physical W/ 5 activity ) OR ( physical W/ 5 function ) OR ( physical W/ 5 performanc* ) OR ( range W/ 5 motion ) OR ( range W/ 5 movement* ) OR flexibili* OR neuromuscular OR balance )) AND PUBYEAR > 2021 AND PUBYEAR < 2025 | **2,242,054** |
|  |  |  |
|  | #24 OR #25 OR #26 OR #27 OR #28 OR #29 OR #30 | **2,421,405** |
|  |  |  |
|  | #10 AND #23 AND #31 | **1,652** |
|  |  |  |
|  | TITLE ( animal* OR invertebrate* OR nonhuman* OR “animal experiment*” OR “animal tissue*” OR “animal model*” OR plant* OR fung* OR agricultural OR cadaver* OR arthroscop* OR surger* OR arthroplast* OR burn* OR thromboemboli* OR fractur* OR stroke OR herbal OR cancer OR latarjet OR bankart OR anchor OR sutur* OR concussi* OR brain OR capsulitis OR frozen OR glycem* OR sclero* OR cortical OR arthrit* OR exoskelet* OR blood* OR reliab* OR valid* OR epidemio* OR clinimetric OR prevalence OR incidence OR “spinal cord” OR wheelchair ) | **6,533,585** |
|  |  |  |
|  | #32 NOT #33 | **1,246** |

**Scopus (Elsevier) - 16/12/22**

|  | TITLE-ABS(“shoulder*”) | **132,755** |
| --- | --- | --- |
|  | TITLE-ABS(“shoulder pain”) | **9,707** |
|  | TITLE-ABS(“shoulder joint”) | **7,342** |
|  | TITLE-ABS(“shoulder injur*”) | **1,786** |
|  | TITLE-ABS(“shoulder impingement”) | **796** |
|  | TITLE-ABS(“subacromial impingement”) | **1,061** |
|  | TITLE-ABS(“subacromial pain”) | **221** |
|  | TITLE-ABS(“rotator cuff”) | **16,602** |
|  | TITLE-ABS(“glenohumeral”) | **8,699** |
|  |  |  |
|  | #1 OR #2 OR #3 OR #4 OR #5 OR #6 OR #7 OR #8 OR #9 | **139,311** |
|  |  |  |
|  | TITLE-ABS(“kinetic chain”) | **1,552** |
|  | TITLE-ABS(“energy transmission”) | **6,007** |
|  | TITLE-ABS(“force transmission”) | **3,275** |
|  | TITLE-ABS(“lower extremit*”) OR TITLE-ABS(“lower limb*”) | **151,973** |
|  | TITLE-ABS(“ankle”) OR TITLE-ABS(“foot”) | **298,546** |
|  | TITLE-ABS(“knee”) | **221,601** |
|  | TITLE-ABS(“hip”) OR TITLE-ABS(“pelvi*”) | **402,038** |
|  | TITLE-ABS(“lumbopelvic”) | **1,097** |
|  | TITLE-ABS(“trunk”) OR TITLE-ABS(“thoracolumbar”) | **119,233** |
|  | TITLE-ABS(“torso”) OR TITLE-ABS(“spine”) | **198,665** |
|  | TITLE-ABS(“thoracic”) OR TITLE-ABS(“core”) | **1,514,761** |
|  | TITLE-ABS(“cervical”) OR TITLE-ABS(“neck”) | **581,069** |
|  |  |  |
|  | #11 OR #12 OR #13 OR #14 OR #15 OR #16 OR #17 OR #18 OR #19 OR #20 OR #21 OR #22 | **3,172,117** |
|  |  |  |
|  | TITLE-ABS(“range of motion”) | **51,790** |
|  | TITLE-ABS(“physical fitness”) | **18,684** |
|  | TITLE-ABS(“motion”) | **1,129,012** |
|  | TITLE-ABS(“muscle strength”) | **33,158** |
|  | TITLE-ABS(“motor”) | **735,477** |
|  | TITLE-ABS(“mobili*”) | **741,132** |
|  | TITLE-ABS ( performanc* OR enduranc* OR ( muscle W/ 5 strength ) OR stabili* OR agili* OR velocit* OR speed* OR power OR ( physical W/ 5 activity ) OR ( physical W/ 5 function ) OR ( physical W/ 5 performanc* ) OR ( range W/ 5 motion ) OR ( range W/ 5 movement* ) OR flexibili* OR neuromuscular OR balance ) | **14,446,591** |
|  |  |  |
|  | #24 OR #25 OR #26 OR #27 OR #28 OR #29 OR #30 | **16,120,507** |
|  |  |  |
|  | #10 AND #23 AND #31 | **10,624** |
|  |  |  |
|  | TITLE ( animal* OR invertebrate* OR nonhuman* OR “animal experiment*” OR “animal tissue*” OR “animal model*” OR plant* OR fung* OR agricultural OR cadaver* OR arthroscop* OR surger* OR arthroplast* OR burn* OR thromboemboli* OR fractur* OR stroke OR herbal OR cancer OR latarjet OR bankart OR anchor OR sutur* OR concussi* OR brain OR capsulitis OR frozen OR glycem* OR sclero* OR cortical OR arthrit* OR exoskelet* OR blood* OR reliab* OR valid* OR epidemio* OR clinimetric OR prevalence OR incidence OR “spinal cord” OR wheelchair ) | **6,085,673** |
|  |  |  |
|  | #32 NOT #33: | **8,631** |

**CINAHL Full Text (EBSCO) - 16/02/24 (2022-2024)**

|  | TI shoulder* OR AB shoulder* | **2,024** |
| --- | --- | --- |
|  | TI shoulder pain OR AB shoulder pain | **409** |
|  | TI shoulder joint OR AB shoulder joint | **130** |
|  | TI shoulder injur* OR AB shoulder injur* | **151** |
|  | TI shoulder impingement OR AB shoulder impingement | **24** |
|  | TI subacromial impingement OR AB subacromial impingement | **20** |
|  | TI subacromial pain OR AB subacromial pain | **28** |
|  | TI rotator cuff OR AB rotator cuff | **568** |
|  | TI glenohumeral OR AB glenohumeral | **201** |
|  |  |  |
|  | S1 OR S2 OR S3 OR S4 OR S5 OR S6 OR S7 OR S8 OR S9 | **2,354** |
|  |  |  |
|  | TI kinetic chain OR AB kinetic chain | **50** |
|  | TI energy transmission OR AB energy transmission | **8** |
|  | TI force transmission OR AB force transmission | **14** |
|  | TI lower extremit* OR TI lower limb* OR AB lower extremit* OR AB lower limb* | **2,435** |
|  | TI ankle OR TI foot OR AB ankle OR AB foot | **4,214** |
|  | TI knee OR AB knee | **5,293** |
|  | TI hip OR TI pelvi* OR AB hip OR AB pelvi* | **6,081** |
|  | TI lumbopelvic OR AB lumbopelvic | **52** |
|  | TI trunk OR TI thoracolumbar OR AB trunk OR AB thoracolumbar | **873** |
|  | TI torso OR TI spine OR AB torso OR AB spine | **1,869** |
|  | TI thoracic OR TI core OR AB thoracic OR AB core | **5,324** |
|  | TI cervical OR TI neck OR AB cervical OR AB neck | **6,879** |
|  |  |  |
|  | S11 OR S12 OR S13 OR S14 OR S15 OR S16 OR S17 OR S18 OR S19 OR S20 OR S21 OR S22 | **28,855** |
|  |  |  |
|  | TI range of motion OR AB range of motion | **1,159** |
|  | TI physical fitness OR AB physical fitness | **466** |
|  | TI motion OR AB motion | **2,356** |
|  | TI muscle strength OR AB muscle strength | **1,213** |
|  | TI motor OR AB motor | **3,543** |
|  | TI mobili* OR AB mobili* | **3,023** |
|  | TI performanc* OR AB performanc* OR TI enduranc* OR AB enduranc* OR TI muscle AND strength OR AB muscle AND strength OR TI stabili* OR AB stabili* OR TI agili* OR AB agili* OR TI velocit* OR AB velocit* OR TI speed* OR AB speed* OR TI power OR AB power OR TI physical AND activity OR AB physical AND activity OR TI physical AND function OR AB physical AND function OR TI physical AND performanc* OR AB physical AND performanc* OR TI range AND motion OR AB range AND motion OR TI range AND movement* OR AB range AND movement* OR TI flexibili* OR AB flexibili* OR TI neuromuscular OR AB neuromuscular OR TI balance OR AB balance | **37,396** |
|  |  |  |
|  | S24 OR S25 OR S26 OR S27 OR S28 OR S29 OR S30 | **42,453** |
|  |  |  |
|  | S10 AND S23 AND S31 | **192** |
|  |  |  |
|  | TI animal* OR TI invertebrate* OR TI nonhuman* OR TI animal experiment* OR TI animal tissue* OR TI animal model* OR TI plant* OR TI fung* OR TI agricultural OR TI cadaver* OR TI arthroscop* OR TI surger* OR TI arthroplast* OR TI burn* OR TI thromboemboli* OR TI fractur* OR TI stroke OR TI herbal OR TI cancer OR TI latarjet OR TI bankart OR TI anchor OR TI sutur* OR TI concussi* OR TI brain OR TI capsulitis OR TI frozen OR TI glycem* OR TI sclero* OR TI cortical OR TI arthrit* OR TI exoskelet* OR TI blood* OR TI reliab* OR TI valid* OR TI epidemio* OR TI clinimetric OR TI prevalence OR TI incidence OR TI 'spinal cord' OR TI wheelchair | **1,116,985** |
|  |  |  |
|  | S32 NOT S33 | **140** |

**CINAHL Full Text (EBSCO) - 16/12/22**

|  | TI shoulder* OR AB shoulder* | **33,114** |
| --- | --- | --- |
|  | TI shoulder pain OR AB shoulder pain | **6,907** |
|  | TI shoulder joint OR AB shoulder joint | **2,255** |
|  | TI shoulder injur* OR AB shoulder injur* | **2,377** |
|  | TI shoulder impingement OR AB shoulder impingement | **757** |
|  | TI subacromial impingement OR AB subacromial impingement | **598** |
|  | TI subacromial pain OR AB subacromial pain | **277** |
|  | TI rotator cuff OR AB rotator cuff | **6,725** |
|  | TI glenohumeral OR AB glenohumeral | **3,360** |
|  |  |  |
|  | S1 OR S2 OR S3 OR S4 OR S5 OR S6 OR S7 OR S8 OR S9 | **36,372** |
|  |  |  |
|  | TI kinetic chain OR AB kinetic chain | **785** |
|  | TI energy transmission OR AB energy transmission | **155** |
|  | TI force transmission OR AB force transmission | **284** |
|  | TI lower extremit* OR TI lower limb* OR AB lower extremit* OR AB lower limb* | **39,871** |
|  | TI ankle OR TI foot OR AB ankle OR AB foot | **66,392** |
|  | TI knee OR AB knee | **76,078** |
|  | TI hip OR TI pelvi* OR AB hip OR AB pelvi* | **101,449** |
|  | TI lumbopelvic OR AB lumbopelvic | **767** |
|  | TI trunk OR TI thoracolumbar OR AB trunk OR AB thoracolumbar | **16,940** |
|  | TI torso OR TI spine OR AB torso OR AB spine | **44,921** |
|  | TI thoracic OR TI core OR AB thoracic OR AB core | **86,392** |
|  | TI cervical OR TI neck OR AB cervical OR AB neck | **113,232** |
|  |  |  |
|  | S11 OR S12 OR S13 OR S14 OR S15 OR S16 OR S17 OR S18 OR S19 OR S20 OR S21 OR S22 | **457,416** |
|  |  |  |
|  | TI range of motion OR AB range of motion | **20,677** |
|  | TI physical fitness OR AB physical fitness | **7,351** |
|  | TI motion OR AB motion | **49,183** |
|  | TI muscle strength OR AB muscle strength | **16,595** |
|  | TI motor OR AB motor | **71,946** |
|  | TI mobili* OR AB mobili* | **49,454** |
|  | TI performanc* OR AB performanc* OR TI enduranc* OR AB enduranc* OR TI muscle AND strength OR AB muscle AND strength OR TI stabili* OR AB stabili* OR TI agili* OR AB agili* OR TI velocit* OR AB velocit* OR TI speed* OR AB speed* OR TI power OR AB power OR TI physical AND activity OR AB physical AND activity OR TI physical AND function OR AB physical AND function OR TI physical AND performanc* OR AB physical AND performanc* OR TI range AND motion OR AB range AND motion OR TI range AND movement* OR AB range AND movement* OR TI flexibili* OR AB flexibili* OR TI neuromuscular OR AB neuromuscular OR TI balance OR AB balance | **634,672** |
|  |  |  |
|  | S24 OR S25 OR S26 OR S27 OR S28 OR S29 OR S30 | **730,933** |
|  |  |  |
|  | S10 AND S23 AND S31 | **3,816** |
|  |  |  |
|  | TI animal* OR TI invertebrate* OR TI nonhuman* OR TI animal experiment* OR TI animal tissue* OR TI animal model* OR TI plant* OR TI fung* OR TI agricultural OR TI cadaver* OR TI arthroscop* OR TI surger* OR TI arthroplast* OR TI burn* OR TI thromboemboli* OR TI fractur* OR TI stroke OR TI herbal OR TI cancer OR TI latarjet OR TI bankart OR TI anchor OR TI sutur* OR TI concussi* OR TI brain OR TI capsulitis OR TI frozen OR TI glycem* OR TI sclero* OR TI cortical OR TI arthrit* OR TI exoskelet* OR TI blood* OR TI reliab* OR TI valid* OR TI epidemio* OR TI clinimetric OR TI prevalence OR TI incidence OR TI 'spinal cord' OR TI wheelchair | **1,042,254** |
|  |  |  |
|  | S32 NOT S33 | **2,927** |
