# Supplementary material for: Thinking outside the shoulder: A systematic review and metanalysis of kinetic chain characteristics in non-athletes with shoulder pain
Source: PLoS One. 2024 Dec 9;19(12):e0314909. doi: 10.1371/journal.pone.0314909 (PMC11627437; doi:10.1371/journal.pone.0314909)
Supplement: S4 File — (DOCX) [file pone.0314909.s004.docx]

**Joanna Briggs Institute (JBI) Critical Appraisal Checklist for Analytical Cross-Sectional Studies**

| STUDY: **Choi & Chung, 2023** | | | | |
| --- | --- | --- | --- | --- |
| **CRITERIA** | **YES** | **NO** | **UNCLEAR** | **NA*** |
| 1. Were the criteria for inclusion in the sample clearly defined? |  |  |  |  |
| 2. Were the study subjects and the setting described in detail? |  |  |  |  |
| 3. Was the exposure measured in a valid and reliable way? |  |  |  |  |
| 4. Were objective, standard criteria used for measurement of the condition? |  |  |  |  |
| 5. Were confounding factors identified? |  |  |  |  |
| 6. Were strategies to deal with confounding factors stated? |  |  |  |  |
| 7. Were the outcomes measured in a valid and reliable way? |  |  |  |  |
| 8. Was appropriate statistical analysis used? |  |  |  |  |
| **OVERALL APPRAISAL (INCLUDE, EXCLUDE, OR SEEK FURTHER INFO): INCLUDE** | | | | |
| ADDITIONAL COMMENTS: none. | | | | |

*NA, not applicable.

**Joanna Briggs Institute (JBI) Critical Appraisal Checklist for Analytical Cross-Sectional Studies**

| STUDY: **Hunter et al., 2020** | | | | |
| --- | --- | --- | --- | --- |
| **CRITERIA** | **YES** | **NO** | **UNCLEAR** | **NA*** |
| 1. Were the criteria for inclusion in the sample clearly defined? |  |  |  |  |
| 2. Were the study subjects and the setting described in detail? |  |  |  |  |
| 3. Was the exposure measured in a valid and reliable way? |  |  |  |  |
| 4. Were objective, standard criteria used for measurement of the condition? |  |  |  |  |
| 5. Were confounding factors identified? |  |  |  |  |
| 6. Were strategies to deal with confounding factors stated? |  |  |  |  |
| 7. Were the outcomes measured in a valid and reliable way? |  |  |  |  |
| 8. Was appropriate statistical analysis used? |  |  |  |  |
| **OVERALL APPRAISAL (INCLUDE, EXCLUDE, OR SEEK FURTHER INFO): INCLUDE** | | | | |
| ADDITIONAL COMMENTS: none. | | | | |

*NA, not applicable.

**Joanna Briggs Institute (JBI) Critical Appraisal Checklist for Analytical Cross-Sectional Studies**

| STUDY: **Maciel & Sousa, 2022** | | | | |
| --- | --- | --- | --- | --- |
| **CRITERIA** | **YES** | **NO** | **UNCLEAR** | **NA*** |
| 1. Were the criteria for inclusion in the sample clearly defined? |  |  |  |  |
| 2. Were the study subjects and the setting described in detail? |  |  |  |  |
| 3. Was the exposure measured in a valid and reliable way? |  |  |  |  |
| 4. Were objective, standard criteria used for measurement of the condition? |  |  |  |  |
| 5. Were confounding factors identified? |  |  |  |  |
| 6. Were strategies to deal with confounding factors stated? |  |  |  |  |
| 7. Were the outcomes measured in a valid and reliable way? |  |  |  |  |
| 8. Was appropriate statistical analysis used? |  |  |  |  |
| **OVERALL APPRAISAL (INCLUDE, EXCLUDE, OR SEEK FURTHER INFO): INCLUDE** | | | | |
| ADDITIONAL COMMENTS: none. | | | | |

*NA, not applicable.

**Joanna Briggs Institute (JBI) Critical Appraisal Checklist for Analytical Cross-Sectional Studies**

| STUDY: **Meurer et al., 2004** | | | | |
| --- | --- | --- | --- | --- |
| **CRITERIA** | **YES** | **NO** | **UNCLEAR** | **NA*** |
| 1. Were the criteria for inclusion in the sample clearly defined? |  |  |  |  |
| 2. Were the study subjects and the setting described in detail? |  |  |  |  |
| 3. Was the exposure measured in a valid and reliable way? |  |  |  |  |
| 4. Were objective, standard criteria used for measurement of the condition? |  |  |  |  |
| 5. Were confounding factors identified? |  |  |  |  |
| 6. Were strategies to deal with confounding factors stated? |  |  |  |  |
| 7. Were the outcomes measured in a valid and reliable way? |  |  |  |  |
| 8. Was appropriate statistical analysis used? |  |  |  |  |
| **OVERALL APPRAISAL (INCLUDE, EXCLUDE, OR SEEK FURTHER INFO): INCLUDE** | | | | |
| ADDITIONAL COMMENTS: none. | | | | |

*NA, not applicable.

**Joanna Briggs Institute (JBI) Critical Appraisal Checklist for Analytical Cross-Sectional Studies**

| STUDY: **Rebelatto et al., 2023** | | | | |
| --- | --- | --- | --- | --- |
| **CRITERIA** | **YES** | **NO** | **UNCLEAR** | **NA*** |
| 1. Were the criteria for inclusion in the sample clearly defined? |  |  |  |  |
| 2. Were the study subjects and the setting described in detail? |  |  |  |  |
| 3. Was the exposure measured in a valid and reliable way? |  |  |  |  |
| 4. Were objective, standard criteria used for measurement of the condition? |  |  |  |  |
| 5. Were confounding factors identified? |  |  |  |  |
| 6. Were strategies to deal with confounding factors stated? |  |  |  |  |
| 7. Were the outcomes measured in a valid and reliable way? |  |  |  |  |
| 8. Was appropriate statistical analysis used? |  |  |  |  |
| **OVERALL APPRAISAL (INCLUDE, EXCLUDE, OR SEEK FURTHER INFO): INCLUDE** | | | | |
| ADDITIONAL COMMENTS: none. | | | | |

*NA, not applicable.

**Joanna Briggs Institute (JBI) Critical Appraisal Checklist for Analytical Cross-Sectional Studies**

| STUDY: **Theisen et al., 2010** | | | | |
| --- | --- | --- | --- | --- |
| **CRITERIA** | **YES** | **NO** | **UNCLEAR** | **NA*** |
| 1. Were the criteria for inclusion in the sample clearly defined? |  |  |  |  |
| 2. Were the study subjects and the setting described in detail? |  |  |  |  |
| 3. Was the exposure measured in a valid and reliable way? |  |  |  |  |
| 4. Were objective, standard criteria used for measurement of the condition? |  |  |  |  |
| 5. Were confounding factors identified? |  |  |  |  |
| 6. Were strategies to deal with confounding factors stated? |  |  |  |  |
| 7. Were the outcomes measured in a valid and reliable way? |  |  |  |  |
| 8. Was appropriate statistical analysis used? |  |  |  |  |
| **OVERALL APPRAISAL (INCLUDE, EXCLUDE, OR SEEK FURTHER INFO): INCLUDE** | | | | |
| ADDITIONAL COMMENTS: none. | | | | |

*NA, not applicable.
